# Robust humoral and cellular recall responses to AZD1222 attenuate breakthrough SARS-CoV-2 infection compared to unvaccinated

**DOI:** 10.3389/fimmu.2022.1062067

**Published:** 2023-01-13

**Authors:** Jill Maaske, Stephanie Sproule, Ann R. Falsey, Magdalena E. Sobieszczyk, Anne F. Luetkemeyer, Grant C. Paulsen, Sharon A. Riddler, Merlin L. Robb, Charlotte-Paige Rolle, Beverly E. Sha, Tina Tong, Bahar Ahani, Anastasia A. Aksyuk, Himanshu Bansal, Timothy Egan, Brett Jepson, Marcelino Padilla, Nirmeshkumar Patel, Kathryn Shoemaker, Ann Marie Stanley, Phillip A. Swanson, Deidre Wilkins, Tonya Villafana, Justin A. Green, Elizabeth J. Kelly

**Affiliations:** ^1^Clinical Development, Vaccines & Immune Therapies, BioPharmaceuticals R&D, AstraZeneca, Gaithersburg, MD, United States; ^2^Biometrics, Vaccines & Immune Therapies, BioPharmaceuticals R&D, AstraZeneca, Gaithersburg, MD, United States; ^3^University of Rochester School of Medicine and Dentistry, Rochester, NY, United States; ^4^Rochester Regional Health, Rochester, NY, United States; ^5^Division of Infectious Diseases, Department of Medicine, Vagelos College of Physicians and Surgeons, New York-Presbyterian Columbia University Irving Medical Center, New York, NY, United States; ^6^Zuckerberg San Francisco General, University of California, San Francisco, San Francisco, CA, United States; ^7^Department of Pediatrics, University of Cincinnati College of Medicine, Cincinnati, OH, United States; ^8^Division of Pediatric Infectious Diseases, Cincinnati Children’s Hospital Medical Center, Cincinnati, OH, United States; ^9^Division of Infectious Diseases, Department of Medicine, University of Pittsburgh, Pittsburgh, PA, United States; ^10^Walter Reed Army Institute of Research, Silver Spring, MD, United States; ^11^Orlando Immunology Center, Orlando, FL, United States; ^12^Division of Infectious Diseases, Department of Internal Medicine, Rush University Medical Center, Chicago, IL, United States; ^13^National Institute of Allergy and Infectious Diseases, National Institutes of Health, Bethesda, MD, United States; ^14^Bioinformatics, Vaccines & Immune Therapies, BioPharmaceuticals R&D, AstraZeneca, Gaithersburg, MD, United States; ^15^Translational Medicine, Vaccines & Immune Therapies, BioPharmaceuticals R&D, AstraZeneca, Gaithersburg, MD, United States; ^16^Clinical Development, Vaccines & Immune Therapies, BioPharmaceuticals R&D, AstraZeneca, Cambridge, United Kingdom

**Keywords:** AZD1222 (ChAdOx1 nCoV-19), COVID-19 vaccine, SARS-CoV-2, breakthrough infection, serology, cell-mediated immunity

## Abstract

**Background:**

Breakthrough severe acute respiratory syndrome coronavirus 2 (SARS-CoV-2) infection in coronavirus disease 2019 (COVID-19) vaccinees typically produces milder disease than infection in unvaccinated individuals.

**Methods:**

To explore disease attenuation, we examined COVID-19 symptom burden and immuno-virologic responses to symptomatic SARS-CoV-2 infection in participants (AZD1222: n=177/17,617; placebo: n=203/8,528) from a 2:1 randomized, placebo-controlled, phase 3 study of two-dose primary series AZD1222 (ChAdOx1 nCoV-19) vaccination (NCT04516746).

**Results:**

We observed that AZD1222 vaccinees had an overall lower incidence and shorter duration of COVID-19 symptoms compared with placebo recipients, as well as lower SARS-CoV-2 viral loads and a shorter median duration of viral shedding in saliva. Vaccinees demonstrated a robust antibody recall response versus placebo recipients with low-to-moderate inverse correlations with virologic endpoints. Vaccinees also demonstrated an enriched polyfunctional spike-specific Th-1-biased CD4+ and CD8+ T-cell response that was associated with strong inverse correlations with virologic endpoints.

**Conclusion:**

Robust immune responses following AZD1222 vaccination attenuate COVID-19 disease severity and restrict SARS-CoV-2 transmission potential by reducing viral loads and the duration of viral shedding in saliva. Collectively, these analyses underscore the essential role of vaccination in mitigating the COVID-19 pandemic.

## Introduction

Vaccination has drastically reduced the global mortality and morbidity burden of coronavirus disease 2019 (COVID-19) (1, 2), yet global circulation of severe acute respiratory syndrome coronavirus 2 (SARS-CoV-2) remains high (3). Breakthrough infections in COVID-19 vaccinees have been observed as immunity to primary series vaccination wanes and new antigenically distinct variants emerge (4–7); however, these typically produce milder disease than infections in unvaccinated individuals (8–11).

AZD1222 (ChAdOx1 nCoV-19) is a simian, replication-deficient, adenovirus-vectored COVID-19 vaccine that has demonstrated safety and efficacy in preventing symptomatic disease (12, 13). To date, the immune response elicited by two-dose primary series AZD1222 vaccination has predominately been studied in SARS-CoV-2-seronegative populations, wherein AZD1222 has been observed to induce systemic anti-SARS-CoV-2 spike glycoprotein (spike), receptor-binding domain, and neutralizing antibody (nAb) responses (13–17), and polyfunctional T helper cell 1 (Th1)-dominated CD4+ and CD8+ cellular immune responses characterized by diverse T-cell receptors with broad coverage of SARS-CoV-2 spike epitopes (14, 18). The protective immune response conferred by AZD1222 vaccination upon breakthrough SARS-CoV-2 infection is less well-characterized.

Prevention of COVID-19 in adults by AZD1222 primary vaccination was studied in a large, diverse population from US, Chile, and Peru in a 2:1 randomized, placebo-controlled, phase 3 study (NCT04516746) (19). Study participants who developed protocol-defined COVID-19 symptoms were requested to contact their study site to initiate illness visits, which entailed collection of nasopharyngeal swabs, saliva samples, sera, and PBMCs for analysis (13). Here, we describe COVID-19 symptom burden and immuno-virologic outcomes in study participants with reverse transcriptase polymerase chain reaction (RT-PCR)-confirmed SARS-CoV-2 infection to characterize the recall response to primary series AZD1222 vaccination and explore disease attenuation compared with placebo recipients.

## Results

### Pooled sera from AZD1222 vaccinees demonstrates a broad neutralizing antibody response against SARS-CoV-2 variants of concern

nAb responses have been proposed as a SARS-CoV-2 correlate of protection across multiple vaccine platforms (20). We have previously analyzed nAb responses against the ancestral SARS-CoV-2 virus 28 days post-second dose primary series in our phase 3 study (13). As outlined in Sobieszczyk et al. (21), data for the follow-up period were obtained prior to and during a global SARS-CoV-2 Alpha wave and Omicron had not been identified at the time of data cut-off (July 30, 2021).

To provide context for this analysis of breakthrough infections, we assessed nAbs elicited against contemporary variants of concern (VoCs) using pooled sera obtained from vaccinees (*n*=210 participants; *n*=21 pools) 28 days following their second dose of primary series AZD1222. These samples were randomly selected from participants enrolled in the immunogenicity substudy of NCT04516746 who had consented to future use of biospecimens for exploratory analyses. Demographics of the participants who contributed to the pools (**Supplemental Table 1**) was representative of the wider NCT04516746 study population (13). nAb geometric mean titers (GMTs) were highest against the ancestral SARS-CoV-2 pseudovirus (202.1), with modest reductions observed against Alpha (133.1), Gamma (59.5), and Delta (103.1) pseudoviruses and larger decreases observed against Beta (32.7) and Omicron BA.1 (21.7) pseudoviruses (**Supplemental Figure 1**).

### Participants with symptomatic SARS-CoV-2 infection

The analyses of SARS-CoV-2 infection in this manuscript are restricted to participants in the fully vaccinated analysis set (FVS) (i.e., those who were SARS-CoV-2 seronegative at baseline and remained on the study for ≥15 days after their second dose of primary series AZD1222 or placebo without SARS-CoV-2 infection) who experienced their first symptomatic RT-PCR-confirmed SARS-CoV-2 infection and provided samples during the 28-day illness period. The overall number of FVS participants who initiated illness visits and had confirmed SARS-CoV-2 positive symptomatic illness confirmed by adjudication was low (AZD1222: *n=*177/17,617; [1.00%]; placebo: *n*=203/8,528, [2.38%]) at the time of data cut-off. The demographics of participants initiating illness visits were broadly similar between AZD1222 and placebo arms (**Supplemental Table 2**). Although vaccinees were younger (median age: 42 versus 46 years, AZD1222 versus placebo), a greater proportion of vaccinees had a very high or high exposure risk to COVID-19 per Occupational Safety and Health Administration categories (27.1% versus 21.2%, AZD1222 versus placebo).

### Illness e-Diary responses illustrate COVID-19 disease attenuation in AZD1222 vaccinees

**Figure 1:**
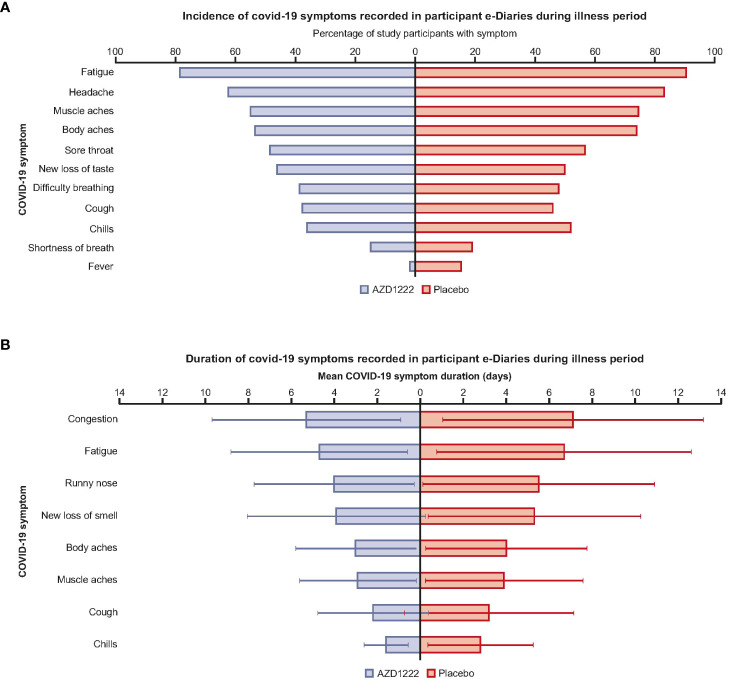
AZD1222 vaccinees had an overall lower incidence and shorter duration of COVID-19 symptoms compared with placebo recipients upon symptomatic SARS-CoV-2 infection. **(A)** Incidence and **(B)** mean duration of self-reported COVID-19 symptoms recorded in participant e-Diaries during the 28-day illness period. **(A)** Symptoms with differences of ≤1.5% between arms were excluded from this plot. **(B)** Symptoms with differences in mean durations of ≤1 day between arms are excluded from this plot. Error bars depict standard deviation.

Participant illness e-Diary responses illustrated that vaccinees had an overall lower incidence of COVID-19 symptoms compared with placebo recipients (**Figure 1A**; **Supplemental Table 3**). Vaccinees had shorter mean symptom durations (by ≥1 day) than placebo recipients for chills (1.6 versus 2.8 days), cough (2.2 versus 3.2), fatigue (4.7 versus 6.7), muscle aches (2.9 versus 3.9), body aches (3.0 versus 4.0), new loss of smell (3.9 versus 5.3), congestion (5.3 versus 7.1), and runny nose (4.0 versus 5.5) (**Figure 1B**; **Supplemental Table 3**). A low incidence of COVID-19 disease in participants aged >65 years limited comparisons regarding symptom incidence and burden between age groups (**Supplemental Table 3**).

### Virologic outcomes are attenuated in AZD1222 vaccinees with breakthrough infections

**Figure 2:**
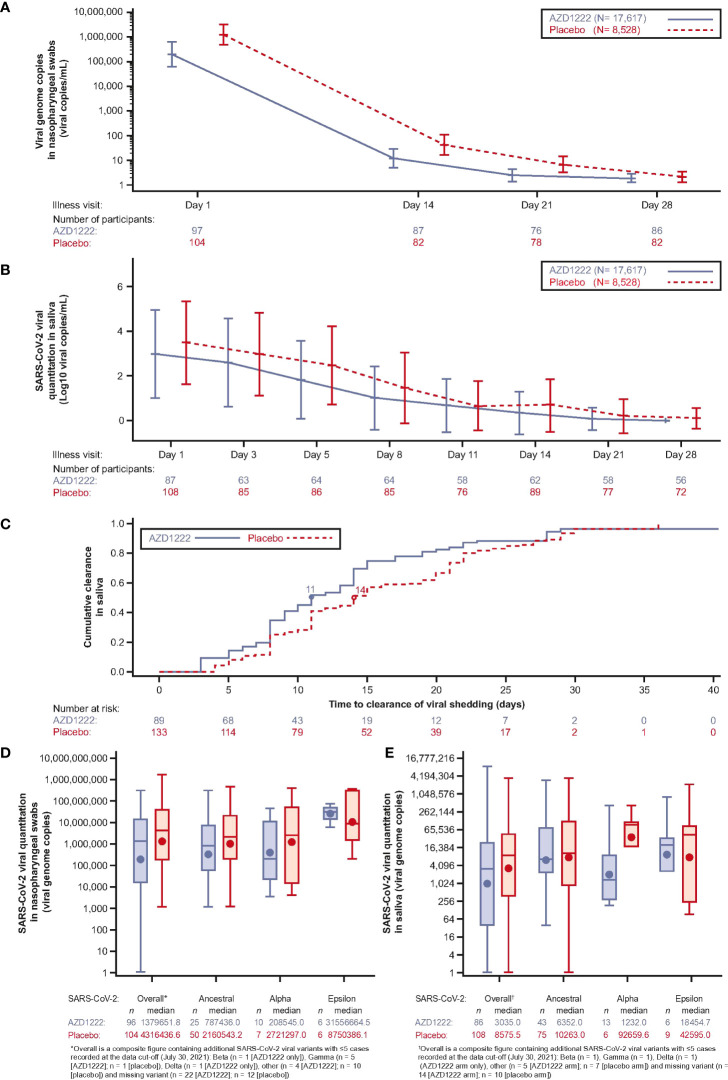
Virologic outcomes to breakthrough infection are attenuated in AZD1222 vaccinees compared to unvaccinated. **(A)** SARS-CoV-2 genome copies from participant nasopharyngeal swabs collected at illness visits determined by quantitative (q)RT-PCR. Line plot with geometric means and 95% CI. Viral genome copies were imputed to 1 when the SARS-CoV-2 nasopharyngeal swab qualitative result was not detected. **(B)** SARS-CoV-2 quantitation (Log10 viral copies/mL) in participant saliva over time. Line plot with mean ± SD. Not detected values of viral quantitation are treated as 0. **(C)** Cumulative incidence plot of SARS-CoV-2 clearance (saliva samples). The median time to clearance of viral shedding for each group is marked by a circle. **(D, E)** Viral load by SARS-CoV-2 variant (viral genome copies) in nasopharyngeal swabs **(D)** and saliva samples **(E)** collected at first illness visit determined by qRT-PCR. The bottom and top edges of the box indicate the first and third quartiles, the difference is the IQR, the line inside the box is the median, and the marker inside the box is the geometric mean. The whiskers that extend from the box indicate the minimum and maximum after removing outliers (i.e., datapoints >1.5 x IQR from the box). Viral genome copies are imputed to 1 when the SARS-CoV-2 nasopharyngeal swab qualitative result is not detected.

Analyses of SARS-CoV-2 viral loads in nasopharyngeal swabs and saliva samples revealed a trend towards lower GMT in vaccinees compared with placebo recipients at all timepoints throughout the illness period (**Figures 2A, B**). The median duration of viral shedding in saliva samples was shortened in vaccinees compared to placebo by 3 days (**Figure 2C**). Among cases, with sequence data at Illness Day 1 (ILL-D1), median overall viral loads in nasopharyngeal swabs (**Figure 2D**) and saliva samples (**Figure 2E**) were lower in vaccinees versus placebo recipients with consistent trends towards lowered viral loads observed for the ancestral SARS-CoV-2 virus and the Alpha variant across both sample types. Median vaccinee SARS-CoV-2 Epsilon viral loads were higher in nasopharyngeal swabs but were lower in saliva samples compared with those observed in placebo.

### AZD1222 vaccinees produce a robust antibody recall response to attenuate SARS-CoV-2 infection

**Figure 3:**
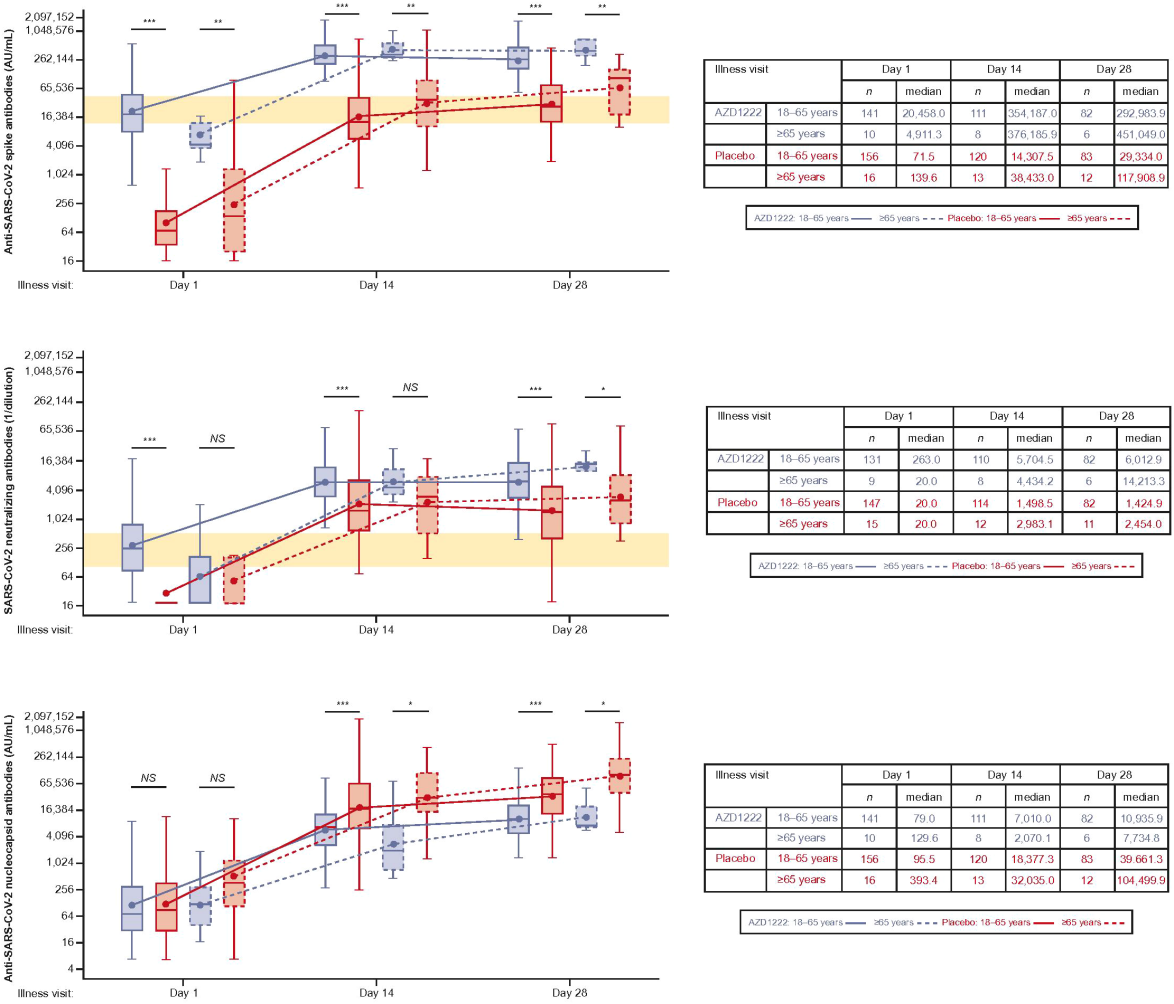
The kinetics and magnitude of the breakthrough anti-SARS-CoV-2 antibody response are impacted by age and vaccination status. Levels of anti-SARS-CoV-2 **(A)** spike-binding, **(B)** neutralizing, and **(C)** nucleocapsid antibodies in AU/mL. The bottom and top edges of the box indicate the first and third quartiles, the difference is the IQR, the line inside the box is the median, and the marker inside the box is the geometric mean. The whiskers that extend from the box indicate the minimum and maximum after removing outliers (i.e., datapoints >1.5 x IQR from the box). **(A, B)** Yellow shaded region denotes peak antibody titers observed during primary analysis [13].Statistical evidence between groups was determined by *post-hoc* two-tailed Mann-Whitney tests. Not significant (NS), p>0.05; *p ≤ 0.05; **p ≤ 0.01; ***p ≤ 0.001.

Median ILL-D1 anti-spike-binding titers in vaccinees were similar to peak titers seen 14 days after dose 2 of AZD1222 (13) and median titers in vaccinees were higher than those observed in placebo recipients at all timepoints (**Figure 3A**). Subgroup analyses illustrated that the kinetics and magnitude of the spike-binding antibody response differed by vaccinee age: median titers were lower at ILL-D1 and higher at ILL-D14 and ILL-D28 in vaccinees aged ≥65 versus those aged 18–64 years, although numbers of participants aged ≥65 years were small (AZD1222: *n*=10; placebo *n=*16).

Median ILL-D1 nAb titers were comparable to peak titers observed 28 days after dose 2 of AZD1222 (13) and further increased throughout the illness visit period, with higher titers in vaccinees than in placebo recipients at all time points (**Figure 3B**). As with anti-spike-binding antibodies, subgroup analyses showed the impact of age on the kinetics and magnitude of nAb response – median titers in vaccinees aged ≥65 versus 18–64 years were lower at ILL-D1 and ILL-D14 but higher at ILL-D28. Median titers within the placebo arm peaked at ILL-D14 and were also higher in participants aged ≥65 versus 18–64 years.

Nucleocapsid antibodies were induced more slowly than spike-binding antibodies and were lower in overall magnitude, with higher median titers in placebo recipients versus vaccinees at ILL-D14 and ILL-D28 (**Figure 3C**). Subgroup analyses revealed median titers were higher in placebo participants aged ≥65 versus 18–64 years. GMTs were broadly similar in vaccinees aged ≥65 or 18–64 years, despite a lower median titer in vaccinees aged ≥65.

**Figure 4:**
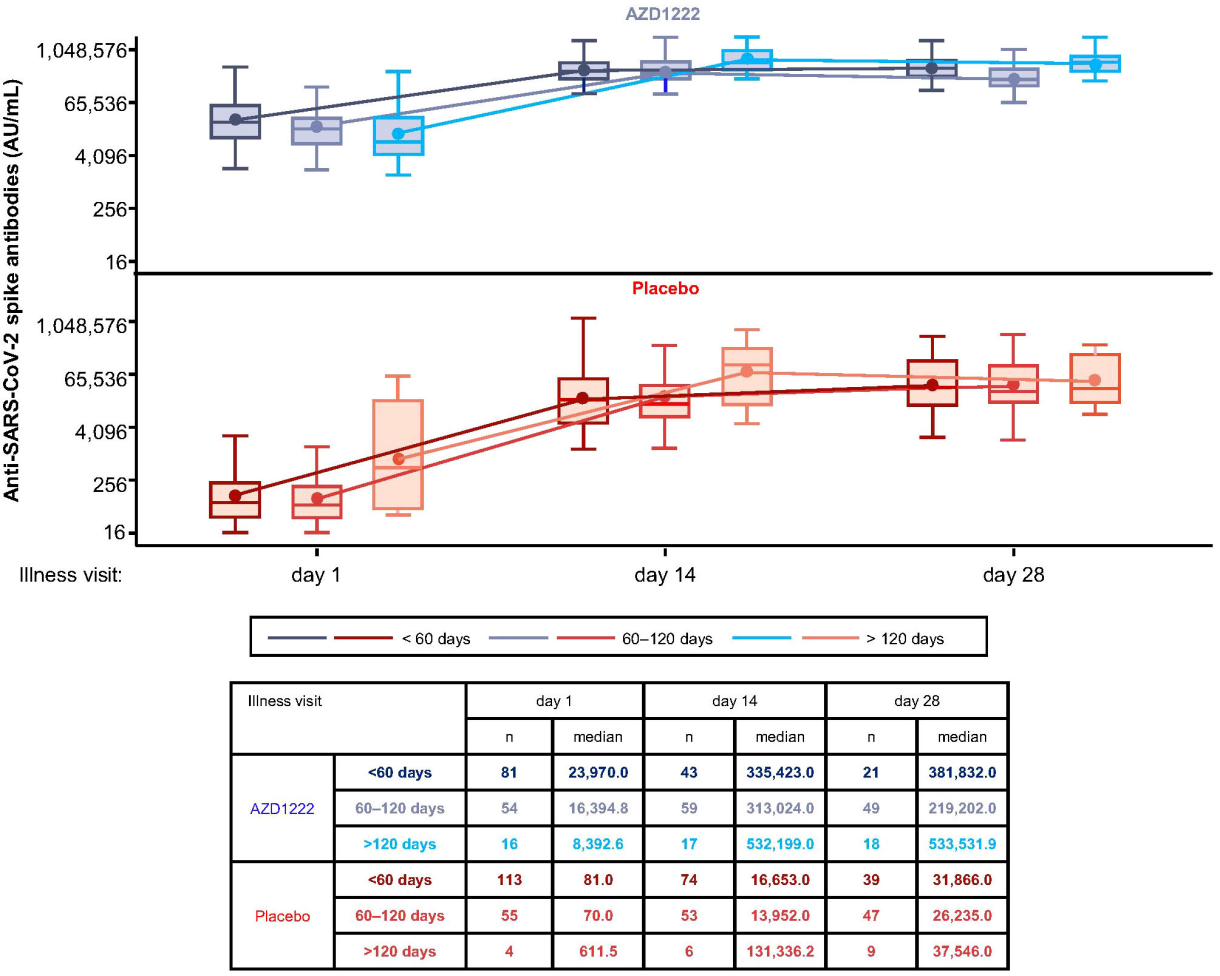
Initial breakthrough SARS-CoV-2 spike-binding antibody titers are influenced by the interval since second dose primary series vaccination. Levels of anti-SARS-CoV-2 spike-binding antibodies by time since second dose of primary series vaccination. The bottom and top edges of the box indicate the first and third quartiles, the difference is the IQR, the line inside the box is the median, and the marker inside the box is the mean. The whiskers that extend from the box indicate the minimum and maximum after removing outliers (i.e., datapoints >1.5 x IQR from the box).

Median spike-binding titers differed with increased time since second dose of primary series vaccination (**Figure 4**). At ILL-D1, lower median spike-binding antibody titers were observed in vaccinees with ≥60 days since second dose primary series than those with <60 days. However, this did not prohibit participants with longer intervals between primary series and illness from mounting equivalent responses to participants with shorter intervals, as evidenced by ILL-D14 and ILL-D28 titers.

**Figure 5:**
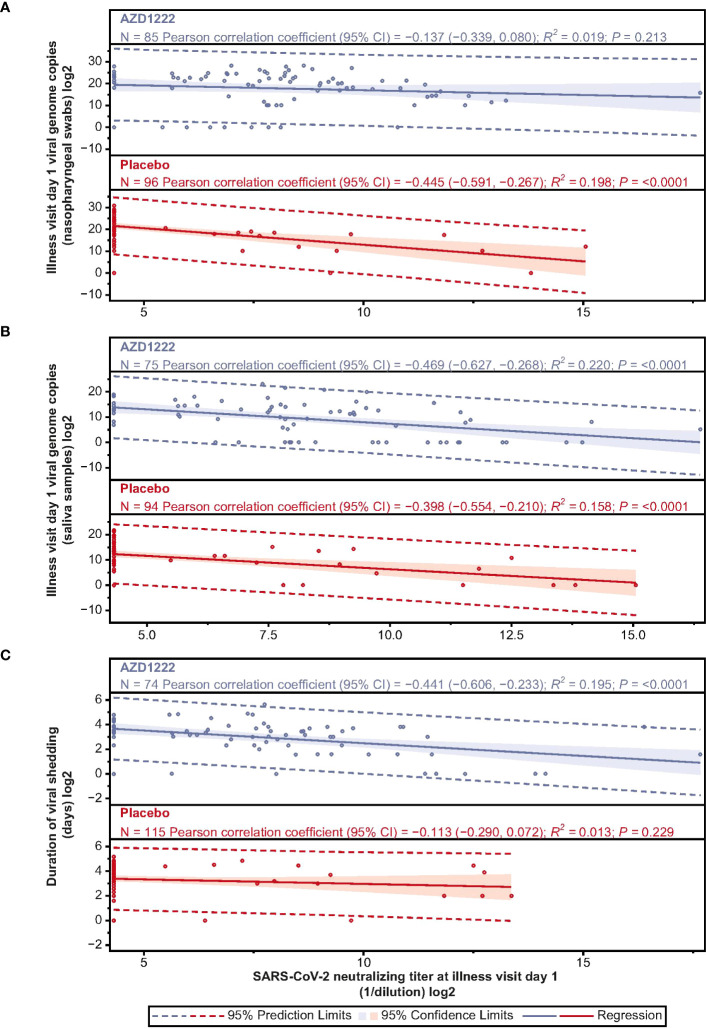
Illness visit day 1 SARS-CoV-2 nAb titers display low-moderate negative correlations with SARS-CoV-2 virologic outcomes. Scatterplot analysis depicting the relationship between **(A)** SARS-CoV-2 viral load in nasopharyngeal swabs **(B)** SARS-CoV-2 viral load in saliva samples, and **(C)** duration of viral shedding in saliva (y-axes) and illness visit day 1 nAb titers (x-axes) in vaccinees and placebo recipients. Blue and red shading denotes 95% CI. Dotted line denotes 95% prediction limits. Clustering of participants along the y-axis occurs due to levels of serum anti-SARS-CoV-2 neutralizing antibody falling below the assay lower limit of quantification (LLOQ). LLOQ= 40 ID50. 50% of LLOQ =20 ID50.

ILL-D1 nAb responses inversely correlated with SARS-CoV-2 virologic outcomes, with low-to-moderate correlations observed for nasopharyngeal viral loads (Pearson: AZD1222 –0.137; placebo –0.445) and viral shedding titers in saliva (Pearson: AZD1222 –0.469; placebo –0.398) (**Figures 5A, B**). ILL-D1 nAb responses in vaccinees displayed moderate correlations with duration of shedding, with weaker correlations observed in placebo recipients (Pearson: AZD1222 –0.441; placebo –0.113) (**Figure 5C**). As outlined in Aksyuk et al. (22), ILL-D1 spike-binding antibody responses also inversely correlated with virologic outcomes, with similar, albeit weaker, low-to-moderate correlations observed for viral shedding titers in saliva (Pearson: AZD1222 –0.436; placebo –0.251). As with ILL-D1 nAb responses, vaccinee spike-binding antibody responses displayed stronger correlations with duration of shedding than those in placebo recipients (Pearson: AZD1222 –0.323; placebo –0.067) (22).

### AZD1222 vaccination induces greater spike-specific T-cell responses following breakthrough infection

**Figure 6:**
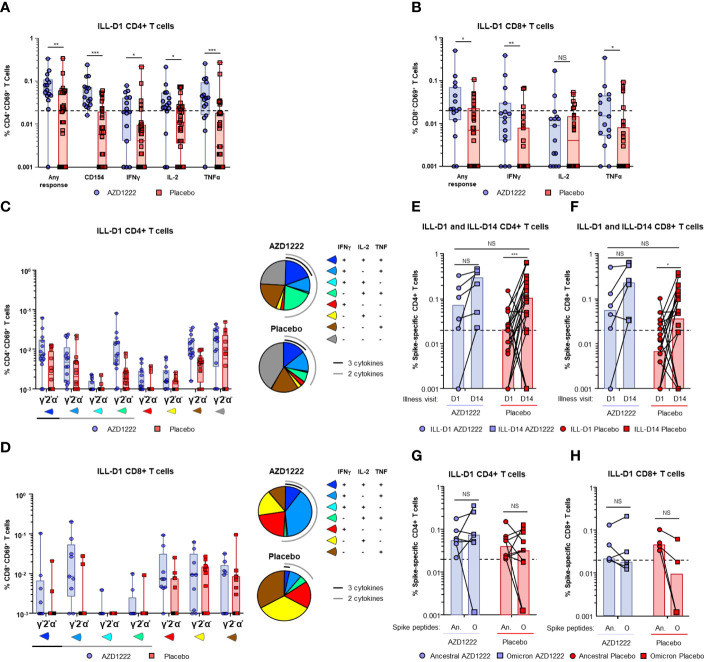
AZD1222 vaccinees possess an enriched SARS-CoV-2 spike-specific T-cell response upon symptomatic SARS-CoV-2 infection compared with placebo recipients. T-cell responses from vaccinees and placebo recipients were assessed following stimulation of PBMCs with SARS-CoV-2 spike peptide pools. Frequencies of SARS-CoV-2 spike protein-specific CD4+ T cells **(A)** expressing CD154, interferon gamma (IFNγ), IL-2, and tumor necrosis factor alpha (TNFα), or any combination of all four (Any Response), and CD8+ T cells **(B)** expressing IFNγ, IL-2, and TNFα, or any combination of all three (Any Response) are shown. Illness visit day 1 cytokine profiles and frequencies of **(C)** CD4+ and **(D)** CD8+ T cell populations upon breakthrough infection in vaccinees and placebo recipients. Spike-specific CD4+ **(E)** and CD8+ **(F)** T-cell frequencies (Any Response) from participants at ILL-D1 and ILL-D14 are shown. Bars indicate median values within each group. Frequencies of spike-specific CD4+ **(G)** and CD8+ **(H)** T-cells (Any Response) against ancestral SARS-COV-2 and Omicron BA.1 variant spike proteins from vaccinee and placebo recipient “responders” at illness visit day 1. In the box and whisker plots the horizontal line represents median, boxes represent IQR, whiskers extend to minimum and maximum, and each symbol represents a participant. Dotted line indicates “responder” threshold. Statistical evidence between groups were determined by two-tailed Mann-Whitney tests. Not significant (NS), P>0.05; *P ≤ 0.05; **P ≤ 0.01; ***P ≤ 0.001. An. = Ancestral; O = Omicron.

Spike-specific CD4+ and CD8+ T-cell responses were assessed by intracellular cytokine staining (ICS) assay following stimulation of participant PBMCs with SARS-CoV-2 spike peptide pools. At ILL-D1, vaccinees had higher frequencies of spike-specific CD4+ (**Figure 6A**) and CD8+ (**Figure 6B**) T cells than placebo recipients (any response CD4+: *P* ≤ 0.01; any response CD8+: *P* ≤ 0.05). Furthermore, AZD1222 vaccination was associated with a higher proportion of responders than placebo recipients (AZD1222 versus placebo: CD4+ 14/15 [93%] versus 16/32 [50%]; CD8+ 10/15 [67%] versus 8/32 [25%]). Interestingly, AZD1222 vaccination did not impact participants *de novo* antiviral T-cell responses as SARS-CoV-2 nucleocapsid-specific CD4+ and CD8+ T-cell frequencies were not statistically different between both groups (**Supplemental Figure 2**).

Spike-specific T cells from responders were further assessed to determine the frequencies and proportions of individual cytokines produced. We found that vaccinees had a greater proportion of polyfunctional Th1 CD4+ T cells (i.e., T cells with the ability to secrete >2 cytokines from the panel) than placebo recipients (**Figure 6C**). Similar to CD4+ T cells, the proportion of polyfunctional spike-specific CD8+ T cells was higher among vaccinees compared to placebo recipients (**Figure 6D**).

At ILL-D14 spike-specific T-cell frequencies and the proportion of responders were increased in both groups (**Supplemental Table 4**), however, elevated responses were generally maintained in vaccinees (**Figures 6E, F**). Although the ancestral strain of SARS-CoV-2 accounted for nearly all breakthrough infections in this analysis, we wanted to determine whether the responders in (**Figures 6A, B**) were also capable of generating a T-cell response to an Omicron VoC lineage BA.1. Stimulation of vaccinee PBMCs with either ancestral SARS-CoV-2 or Omicron BA.1 spike peptide pools revealed equivalent CD4+ and CD8+ T-cell responses (**Figures 6G, H**) indicating that AZD1222 vaccination induces broad recognition of the SARS-CoV-2 spike protein as previously demonstrated (18). T cells from placebo participants were also able to recognize Omicron BA.1 spike peptides indicating natural infection can also induce a broad T-cell response.

**Figure 7:**
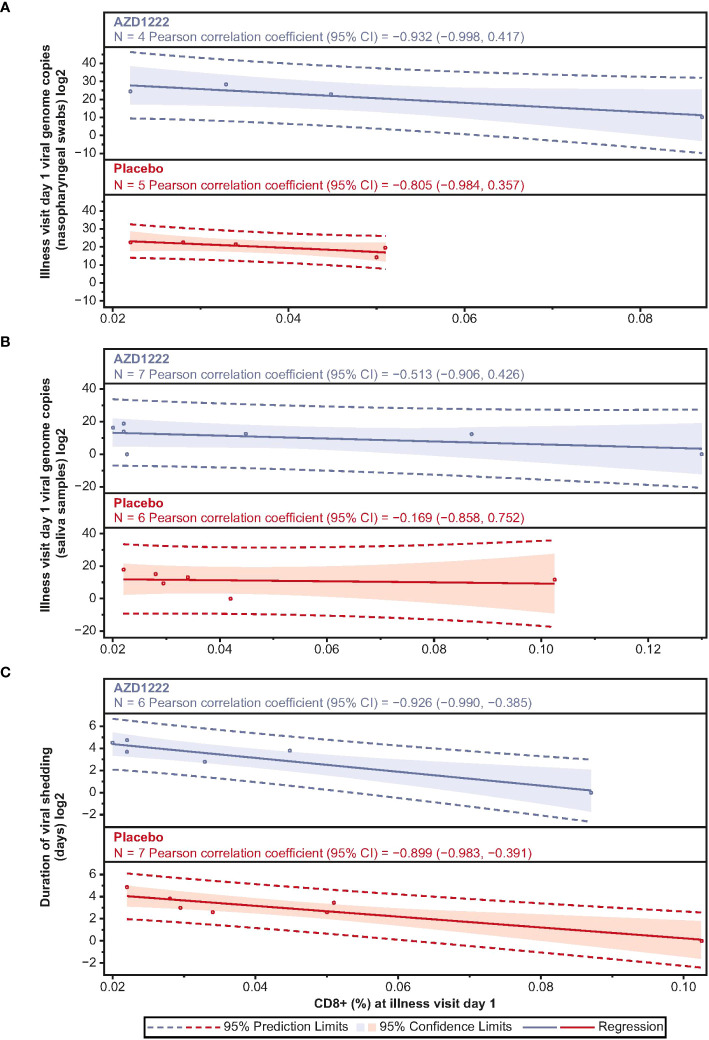
Illness visit day 1 CD8+ titers display strong negative correlations with SARS-CoV-2 virologic outcomes. Scatterplot analysis depicting the relationship between **(A)** SARS-CoV-2 viral load in nasopharyngeal swabs **(B)** SARS-CoV-2 viral load in saliva samples, and **(C)** duration of saliva viral shedding (y-axes) and illness visit day 1 spike-specific CD8+ T cell frequencies (x-axes) in vaccinees and placebo recipients. Blue and red shading denotes 95% confidence limits. Dotted line denotes 95% prediction limits.

Correlations between ILL-D1 T-cell and humoral responses and virologic outcomes were assessed. ILL-D1 nAb titers in vaccinees directly correlated with CD4+ (Spearman rank: 0.65; *p*=0.02) but not CD8+ (Spearman rank: 0.05; *p*=0.85) T-cell responses (**Supplemental Figure 3**). Among participants with matched PBMCs and virology samples, CD4+ T-cell responses inversely correlated with nasopharyngeal viral loads with strong correlations observed in vaccinees (Pearson: AZD1222 –0.881; placebo 0.796) (**Supplemental Figure 4**). However, trends were inconsistent between groups for durations of shedding (Pearson: AZD1222 –0.421; placebo –0.344) and shedding titers in saliva (Pearson: AZD1222 –0.681; placebo 0.126). CD8+ T-cell responses also inversely correlated with virologic outcomes, with strong correlations observed with quantitative viral loads (Pearson: AZD1222 –0.932; placebo –0.805), and the duration of shedding (Pearson: AZD1222 –0.926; placebo –0.899) in both groups (**Figure 7**). Similar to CD4+ T cells, moderate correlations were observed between ILL-D1 CD8+ T-cell responses and viral shedding titers in saliva (Pearson: AZD1222 –0.513; placebo –0.169) for vaccinees but not placebo recipients. However, the numbers of data points were limited in these analyses and findings should be interpreted accordingly.

## Discussion

The immune response to COVID-19 vaccination has been extensively studied in SARS-CoV-2-seronegative populations during randomized placebo-controlled studies (23). Beyond their immediate implications for further immunization campaigns, studies of immune responses to breakthrough SARS-CoV-2 infection are invaluable due to the insights they can provide for infection control, disease attenuation, and the design of future COVID-19 vaccines (24). In this context, we have comprehensively evaluated immuno-virologic outcomes in symptomatic baseline-seronegative vaccinees and unvaccinated controls to characterize the recall response to AZD1222 vaccination. Our findings are strengthened by our sample size (N=380) and the range of paired virologic, serologic, and cellular outcomes assessed in light of self-reported COVID-19 symptoms. Collectively, these data provide an invaluable insight into crucial aspects of effective SARS-CoV-2 immunity.

Retrospective case studies (8, 9) and real-world symptomology studies (10) have shown a reduced COVID-19 disease burden in vaccinees upon breakthrough infection. Our dataset suggests a similar trend towards disease attenuation; illness e-Diary responses illustrate that vaccinees experienced fewer and a shorter duration of COVID-19 symptoms – particularly for systemic symptoms such as chills, fatigue, muscle aches, and body aches – compared with unvaccinated participants. A lower magnitude and quicker decline of SARS-CoV-2 viral load has previously been linked with reduced COVID-19 disease severity (25, 26). We observed lower mean viral loads in vaccinee nasopharyngeal swabs and saliva samples compared with those from placebo recipients across all illness visit timepoints and a shortened duration of viral shedding, supporting the symptomologic profile of disease attenuation in vaccinees. Viral loads in samples from early illness visits were lower in vaccinees versus placebo recipients overall, and for the ancestral SARS-CoV-2 virus and the Alpha variant. Collectively, these data suggest that the recall response following AZD1222 vaccination enables vaccinees to exert quicker and overall greater control of breakthrough infections than the unvaccinated and are consistent with similar analyses of breakthrough infection in other COVID-19 vaccinees (11, 27, 28).

ILL-D1 spike-binding and nAb titers in vaccinees were comparable to peak titers observed post-primary series (13). Median spike-binding and nAb titers were higher than those observed in placebo recipients across all timepoints as documented in other serological studies of breakthrough infection (29, 30). We observed differences in the kinetics and magnitude of anti-SARS-CoV-2 nucleocapsid antibody responses between vaccinees and placebo recipients. We hypothesize these differences may be due to effective anti-spike memory responses resulting in lower viral loads in vaccinees later in the illness period, thus attenuating their exposure to nucleocapsid antigen compared with placebo recipients. Additionally, spike-specific memory B cells would be more numerous than naïve lymphocytes in vaccinees and would be activated by lower antigenic thresholds with affinity matured memory B cells potentially outcompeting naïve B cells for T follicular helper cell stimulation (31, 32).

Real-world serological studies have demonstrated that spike-binding antibody titers begin to wane as early as 4–6 months post-primary series vaccination (33, 34). ILL-D1 spike antibody titers were lower in vaccinees who were 60–120 days and >120 days post-second dose primary series. However, immunological waning did not affect the overall magnitude or kinetics of the recall response following AZD1222 vaccination, with similar overall responses seen by the end of the illness period regardless of interval since primary series vaccination – potentially enabling longer intervals between booster doses. These data could provide important insights to inform policy decisions on further immunization campaigns.

ILL-D1 spike-binding and nAb IgG responses inversely correlated with virologic outcomes, with moderate correlations observed for viral shedding titers and for the duration of shedding in saliva – particularly in vaccinees. As SARS-CoV-2 is transmitted *via* salivary droplets (35), these findings underscore the importance of vaccination in limiting onward transmission. Induction of oronasal anti-spike IgM, IgG, and IgA has been observed following natural SARS-CoV-2 infection (36–41), with several studies suggesting that secretory IgA levels inversely correlate with susceptibility to breakthrough infection (30, 40, 42). We have previously examined nasal immunogenicity following AZD1222 vaccination in a separate exploratory analysis (22), therein we observed that intramuscular AZD1222 vaccination elicits anti-spike IgG responses in nasal epithelial lining fluid (NELF), likely reflecting transudation of serum IgG to the nasal mucosa. AZD1222 vaccinees who experienced breakthrough infections displayed robust anamnestic IgG responses, which correlated with reduced viral loads and durations of viral shedding in saliva. Although we and others have observed transient increases in NELF IgA levels from vaccinees with prior SARS-CoV-2 infection (22, 41, 43), intramuscular vaccination with adenovirus- and mRNA-based COVID-19 vaccines does not appear to induce anamnestic NELF IgA responses in SARS-CoV-2-seronegative vaccinees despite eliciting IgA responses in serum (43–45). These findings suggest that different approaches (e.g., use of adjuvants, other delivery routes) will be required to improve mucosal immunogenicity for currently licensed COVID-19 vaccines (46). nAb titers have previously been proposed as a SARS-CoV-2 correlate of protection based on the reduced vaccine efficacy observed against symptomatic Beta and Omicron VoC infection, the restoration of waning vaccine efficacy by booster doses, and insights gained from serological studies of convalescent individuals and COVID-19 vaccinees (47–53). *In vitro* serum testing demonstrated the breadth of nAb response elicited by primary series AZD1222 vaccination across contemporary VoC with expected reductions for SARS-CoV-2 Beta and Omicron variants (54). These nAb evasive properties, coupled with waning immunity over time, could lead to an increased frequency of breakthrough infections mediated by Omicron. However, studies of related coronaviruses SARS-CoV-1 and Middle East respiratory syndrome coronavirus (MERS-CoV), and the enduring protection against Beta- and Omicron-mediated severe disease in vaccinees (6, 55), suggest that the cellular immune response is an equally important mediator of COVID-19 disease severity (56–59).

Frequencies of spike-specific CD4+ and CD8+ T cells were enhanced in vaccinees compared to placebo recipients at ILL-D1, likely due to pre-existing memory cells in vaccinees. Surprisingly, these enhanced responses were maintained 2 weeks post-ILL-D1. It is possible that vaccinee spike-specific memory T cells continued to expand over this period, however, it is also likely that the addition of *de novo* spike-specific CD4+ and CD8+ T cells contributed to this disparity. We demonstrated that at ILL-D1 nucleocapsid-specific CD4+ and CD8+ T cell responses were equivalent between vaccinees and placebo recipients indicating that AZD1222 vaccination did not impact the *de novo* antiviral response. As studies of SARS-CoV-1 suggest that nucleocapsid-specific T-cell responses can persist for up to 17 years post-infection, and can potentially provide protection against other *betacoronaviruses* (57), it is encouraging that vaccinees’ nucleocapsid-specific T-cell response is not majorly impeded by prior spike-directed immunological memory. Therefore, AZD1222 vaccination leads to a combined anamnestic and *de novo* T cell response which gives rise to faster, stronger antiviral immunity following breakthrough infection.

Vaccinee T cells displayed a greater proportion of polyfunctional markers compared with placebo recipients. Polyfunctional T cells are most abundant in individuals with mild COVID-19 (25) and are proposed to impede the development of moderate and severe disease. Curiously, a proportion of spike-specific CD4+ T cells in vaccinees and placebo recipients expressed CD154+ in the absence of canonical Th1 cytokines (although the proportion was higher in placebo recipients) indicating that they may perform other undetected antiviral effector functions such as cytotoxicity (60, 61).

One limitation to this study is that data were obtained prior to and during a global SARS-CoV-2 Alpha variant wave and thus breakthrough infections were caused by circulating SARS-CoV-2 variants that are no longer VoCs. Would the vaccinees have been imparted with a similar antiviral cellular advantage had they been infected by an Omicron variant? We and others have observed that primary series AZD1222 vaccination induces durable, diverse T-cell responses with minimal viral escape from VoC including Omicron BA.1 (18, 62). However, it was still unknown whether these diverse T-cell responses were maintained following breakthrough infection. In this study we showed that spike-specific T cells from vaccinees can recognize ancestral and Omicron BA.1 variants equivalently.

Cellular immune responses inversely correlated with virologic outcomes, which is in line with observations that effective viral clearance by cellular immunity correlates with milder COVID-19 disease severity (63, 64). Stronger correlations with reduced viral titers in saliva were observed in vaccinees compared with placebo recipients, underscoring the role of vaccination in limiting onward SARS-CoV-2 transmission. Our data supports the hypothesis that ILL-D1 T-cell responses display stronger correlations with virologic outcomes than nAb levels due to differential kinetics of the cellular and humoral response following breakthrough infection as demonstrated by the high level of ILL-D1 CD4+ and CD8+ T-cell response observed in vaccinees compared with placebo (CD4+ 93% versus 50%; CD8+ 67% versus 25%). Others have observed CD8+ T cells as early as 1 day post-symptom onset in unvaccinated individuals (65) and the rapid induction of CD4+ and CD8+ T-cell responses within 1 week of symptom onset has been associated with milder COVID-19 disease (66). Preliminary estimates of the longevity of cell-mediated immunity to SARS-CoV-2 have been documented by others following vaccination (67) and natural infection (68, 69). Although the longevity of cell-mediated immunity to SARS-CoV-2 is currently undetermined, T cells from convalescent individuals following SARS-CoV-1 (57) MERS-CoV (56) infection suggest that cellular responses are robust and can persist for several years following infection.

Limitations of this analysis include the low numbers of participants >65 years of age who experienced breakthrough infection, and the small sample size for the T-cell correlative analyses. As described in Sobieszczyk et al. (21), we have previously observed evidence of under-reporting of non-study COVID-19 vaccination in the placebo arm. As participants aged ≥65 were among the first groups who were eligible to receive a non-study COVID-19 vaccination, this may in part explain the increased spike-binding and nAb responses we observed in this cohort of the placebo group during the serology analyses.

In summary, breakthrough infection in AZD1222 vaccinees was characterized by a lower symptom burden, lower viral loads, and more robust humoral and cellular responses compared with unvaccinated participants. Our findings are intriguing in light of other studies with vaccinees who received primary series mRNA-based COVID-19 vaccines, wherein a similar attenuated disease profile with reduced viral loads (11, 27, 28) and robust humoral (29, 30, 70) and cellular immune responses (70–72) have also been observed following breakthrough infection. While direct comparisons between studies is confounded by differences in time since breakthrough infection, circulating VoCs, and study design, these studies demonstrate a consistent pattern of disease attenuation in vaccinated individuals.

Although sample numbers were limited, cellular immune responses displayed strong inverse correlations with viral endpoints upon breakthrough infection, emphasizing the importance of cellular immunity in protective immune responses against COVID-19. Limiting SARS-CoV-2 viral transmission is a key step to overcoming the COVID-19 pandemic. Our data also indicate that the induction of T-cell recall responses early following breakthrough correlated with reduced viral loads in saliva compared to unvaccinated individuals. Of note, COVID-19 vaccines designed using the ancestral SARS-CoV-2 spike protein continue to confer protection against severe disease, likely arising from the conservation of T-cell epitopes in previous and contemporary VoCs (62, 73–77). This enduring protection – despite an increase in breakthrough infections – has led to a new era of “hybrid immunity”, which will have ramifications for future immunization strategies as new antigenically distinct SARS-CoV-2 VoCs continue to evolve (78). Collectively, these observations underscore the central and essential role of vaccination in attenuating COVID-19 disease, limiting onward SARS-CoV-2 transmission, and mitigating the COVID-19 pandemic.

## Materials and methods

### Study design

As previously reported (13, 21), NCT04516746 was designed as a double-blind, placebo-controlled, phase 3 study of the safety and efficacy of 28-day primary series AZD1222 for the prevention of symptomatic COVID-19 in participants ≥18 years of age whose conditions were medically stable and who were at increased risk for SARS-CoV-2 infection. Participants were recruited from 88 sites in the United States, Chile, and Peru. The study was conducted in accordance with the principles of the Declaration of Helsinki and the International Council for Harmonization Good Clinical Practice guidelines. All participants provided written informed consent before enrollment.

Study participants were randomly assigned in a 2:1 ratio to receive two injections of AZD1222 (5×10^10^ viral particles), or saline placebo administered 4 weeks apart on days 1 and 29 (−3 to +7 days). Randomization was stratified according to age (≥18–64 years and ≥65 years), with a target of ≥25% participants being ≥65 years of age.

### Qualifying COVID-19 symptoms for initiating illness visits

Study participants who experienced any duration of fever, shortness of breath, difficulty breathing, ≥2 days of chills, cough, fatigue, muscle aches, body aches, headache, new loss of taste, new loss of smell, sore throat, congestion, runny nose, nausea, vomiting, or diarrhea were requested to contact their study site to initiate illness visits (13, 21).

### Illness visits

All participants with qualifying symptoms underwent an initial illness visit for confirmatory SARS-CoV-2 RT-PCR testing and provided nasopharyngeal swabs, self-collected saliva samples (US-sites only), sera, and PBMCs (select-sites only) for analysis. Participants were also trained to operate an illness e-Diary to document their symptoms (**Supplemental Methods**). Only participants with confirmed SARS-CoV-2 infection continued the full 28-day illness visit course, which comprised an “at home” period with self-collection of saliva samples on days 3, 5, 8, and 11, and additional site-visits with collection of nasopharyngeal swabs, saliva samples, sera, and PBMCs (day 14 illness visit only) on days 14, 21, and 28. Some saliva samples were collected at home based upon investigator preference. If a participant had multiple sets of illness visits, the first set of illness visits with positive RT-PCR test result was used for the summary.

### Illness e-Diary responses

Participants were trained by site staff on how to record their symptoms in an illness e-Diary during the day 1 illness visit. Participants who tested positive for SARS-CoV-2 continued recording their symptoms until symptom resolution or until the day 28 illness visit. Site staff monitored the health status of participants *via* Illness e-Diary responses and called participants as needed based on their responses.

### Illness visit sample availability

Not all participants contributed data at every illness visit. Of the 380 participants (AZD1222, *n*=177; placebo, *n*=203) with symptomatic infection, nasopharyngeal swabs, and saliva samples from 201 (AZD1222, *n*=97; placebo, *n*=104) and 222 (AZD1222, n=89; placebo, *n*=133) participants, respectively, were available for virologic assessments, sera from 323 (AZD1222, *n*=151; placebo, *n*=172) participants were analyzed for anti-SARS-CoV-2 serologic responses, and PBMCs from 47 (AZD1222, *n*=15; placebo, *n*=32) participants were analyzed by ICS to evaluate SARS-CoV-2 spike-specific CD4+ and CD8+ T-cell responses.

### SARS-CoV-2 virologic assessments

As previously described (13, 21), SARS-CoV-2 viral load was assessed in nasopharyngeal swabs and saliva samples using the TaqPath™ SARS-CoV-2 RT-PCR Assay (ThermoFisher Scientific, Waltham, MA, USA). Nasopharyngeal swabs were analyzed by Labcorp, Indianapolis, IN, USA, while saliva samples were analyzed by Infinity Biologix, Rutgers, NJ, USA. SARS-CoV-2 genomic assessments are detailed in full in **Supplemental Methods**. Assessments of viral titer and sequencing were only performed on participants from whom sample was available after the completion of a central RT-PCR assay, thus limiting the availability for results in all participants.

### Anti-SARS-CoV-2 serology analyses

Serum anti-SARS-CoV-2 spike-binding and nucleocapsid IgG antibody titers were tested at PPD^®^ in a validated multiplex electrochemiluminescence serology assay using the MSD V-PLEX^®^ SARS-CoV-2 Panel 2 (IgG) as outlined in (79). nAbs were assessed in a validated lentivirus-based SARS-CoV-2 phenosense pseudovirus assay (Monogram Biosciences, South San Francisco, CA, USA) as described previously (13). nAbs titers are reported as the reciprocal of the serum dilution conferring ID_50_ of pseudovirus infection.

Formulae to enable conversion from arbitrary units per milliliter (AU)/mL to the WHO international standard (National Institute for Biological Standards and Control [NIBSC] 20/136) binding units (BAU/mL) and ID_50_ to the WHO international standard (NIBSC 20/136) International units (IU/mL) are detailed in **Supplemental Methods**.

### T-cell stimulation and analysis

An ICS assay was used to evaluate T-cell responses, as previously described (18). Please refer to **Supplemental Methods** for full details on T cell stimulation with SARS-CoV-2 spike peptides, ICS antibody staining, and the flow cytometry gating strategy used in these analyses.

### Statistics

The analyses presented in this manuscript are restricted to baseline-seronegative participants with PCR-confirmed SARS-CoV-2 infection ≥15 days after dose 2 of AZD1222 or placebo per the protocol definition of breakthrough infection (13). Definitions of the study populations (i.e., the FVS and immunogenicity analysis set [IAS]) used to compile the participant demography (**Supplemental Table 2**) and participants with illness visits e-Diary data (**Figure 1**; **Supplemental Table 3**) tables are included in **Supplemental Material**. For ethical reasons, study participants could be unblinded and receive non-study COVID-19 vaccinations once available through emergency-use authorizations. The censoring implications of allowing participants to receive non-study COVID-19 vaccinations are detailed in **Supplemental Methods** GMTs were calculated and summarized at each illness visit for viral load assessments in nasopharyngeal swabs and saliva samples, and for anti-SARS-CoV-2 spike-binding, neutralizing and nucleocapsid antibodies in AZD1222 and placebo groups. SARS-CoV-2 spike-binding, neutralizing, and nucleocapsid antibodies were also assessed by participant age and time since second dose of primary series. GMT endpoints were analyzed on the natural log scale by separate ANOVA models including treatment and age as categorical covariates. On the log scale, the models were used to estimate a mean response for the vaccine and control groups and the difference (vaccine – control), with corresponding 95% confidence limits. Descriptive statistics for GMTs included the number of participants, geometric mean, 95% CI, minimum, and maximum. A GMT was calculated as the antilogarithm of Σ(log base 2-transformed titer/n), i.e., as the antilogarithm transformation of the mean of the log-transformed titer, where *n* is the number of participants with titer information. The 95% CI was calculated as the anti-logarithm transformation of the upper and lower limits for a two-sided CI for the mean of the log-transformed titers.

SAS 9.4 procedure SGPANEL was used to create the scatter plots for the correlative analyses. The REG statement generated the fitted regression line along with confidence limit intervals (CLI) and confidence limit for the mean (CLM) options to create the prediction limits and confidence limits respectively.

Informal comparisons between groups were done by *post-hoc* two-tailed Mann-Whitney tests and categorized as: not significant (NS), p>0.05; *p ≤ 0.05; **p ≤ 0.01; ***p ≤ 0.001.

### Study approval

The protocol and amendments for this trial (ClinicalTrials.gov number, NCT04516746) were approved by the ethics committee or institutional review board at each center, and the trial was conducted in compliance with the principles of the Declaration of Helsinki and the International Council for Harmonization Good Clinical Practice guidelines. Prior to enrolment all participants provided informed consent.

## Data availability statement

Data associated with this study are available in the main text or the supplementary materials, excluding data underlying the clinical findings. Data underlying the clinical findings described in this manuscript may be requested in accordance with AstraZeneca’s data sharing policy described at https://astrazenecagrouptrials.pharmacm.com/ST/Submission/Disclosure. AstraZeneca Group of Companies allows researchers to submit a request to access anonymized patient level clinical data, aggregate clinical or genomics data (when available), and anonymized clinical study reports through the Vivli web-based data request platform.

## Ethics statement

The studies involving human participants were reviewed and approved by the ethics committee or institutional review board at each participating center, and the trial was conducted in compliance with the principles of the Declaration of Helsinki and the International Council for Harmonization Good Clinical Practice guidelines. The patients/participants provided their written informed consent to participate in this study.

## Author contributions

Design of study: ARF, MES, TV, JAG, and EJK in collaboration with the US Government and the sponsor; Collection of samples: ARF, MES, AFL, GCP, SAR, MLR, C-PR, and BES; Analysis of PRO data: JM, SS, and EJK; Analysis of nasopharyngeal swabs, saliva, and serum samples: AAA, AMS, DW, JM, SS, and EJK; Analysis of viral sequences: BA; Analysis of peripheral blood mononuclear cells: MP, PAS, JM, SS, and EJK; Statistical programming/correlative analyses: TT, HB, TE, NP, BJ, KS, and SS; Confirmation of data accuracy: HB, SS, PAS, JM, and EJK. All authors reviewed and provided substantive revisions to subsequent drafts, and all authors approved the final draft and the decision to submit for publication. Data collection, sample analyses, and generation of analysis datasets were supported by IQVIA, a contract research organization.
