# Supplementary material for: Robust humoral and cellular recall responses to AZD1222 attenuate breakthrough SARS-CoV-2 infection compared to unvaccinated
Source: Front Immunol. 2023 Jan 13;13:1062067. doi: 10.3389/fimmu.2022.1062067 (PMC9881590; doi:10.3389/fimmu.2022.1062067)
Supplement: Supplementary file 1 [file DataSheet_1.docx]

**List of Supplementary Materials**

Materials and Methods

Supplemental Figure 1. nAb responses 28 days post-AZD1222 primary-series vaccination are consistent with expected immune-evasive properties of SARS-CoV-2 Variants of Concern.

Supplemental Figure 2. Nucleocapsid-specific CD4+ and CD8+ T cells responses in AZD1222 vaccinees and placebo recipients.

Supplemental Figure 3. Illness visit day 1 CD4+ T-cell responses correlate with Illness Day 1 nAb titers.

Supplemental Figure 4. Illness visit day 1 CD4+ T cell levels moderately correlate with SARS-CoV-2 virologic outcomes.

Supplemental Table 1. Demographics of fully vaccinated participants who provided serum samples for exploratory immunogenicity analyses.

Supplemental Table 2. Demographics of participants with illness visits (fully vaccinated analysis set).

Supplemental Table 3. Incidence and duration of self-reported COVID-19 symptoms.

Supplemental Table 4. Frequencies of spike-specific CD4+ and CD8+ T-cell responses at ILL-D1 and ILL-D14.

Supplemental Table 5. Amino acid changes incorporated into variant of concern pseudoviruses.

Supplemental Table 6 List of antibodies used for intracellular cytokine staining.

**Methods**

**Participant selection for pooled vaccinee serum pools**

Serum samples were obtained from 210 participants 28 days following their second dose of primary series AZD1222, who had consented to future use of biospecimens and were selected randomly from participants with >2 mLs of serum available. Twenty one pools were created by equally pooling 1 mL of serum from 10 study participants. The demography of the selected pools was representative of the NCT04516746 study population; the median age was 52.5 years and 52.9% of study participants reported at least one coronavirus disease 2019 (COVID-19) comorbidity (Supplemental Table 1). Female participants were overrepresented in these pools as compared to the overall phase 3 study population from which they were derived with 63.8% of participants being female.

**Pseudovirus neutralization assay**

Neutralizing antibody (nAb) titers were assessed using the PhenoSense severe acute respiratory syndrome coronavirus 2 (SARS-CoV-2) nAb assay by generating HIV-1 pseudovirions that express the SARS-CoV-2 spike protein and measuring the inhibition of luciferase activity in HEK293 target cells expressing the ACE2 receptor, following pre-incubation of the pseudovirions with serial dilutions of study participant serum. The expression of luciferase activity in target cells is inhibited in the presence of anti-SARS-CoV-2 neutralizing antibody.

nAb titers are reported as the ID_50_ of pseudovirus infection:

$$\%Inhibition =$$

$$100\% - (((RLU(Vector+Sample+Diluent) -RLU(Background))$$

$/$(RLU(Vector+Diluent) – RLU(Background))) x 100%)

To ensure that the nAb activity is SARS-CoV-2 specific, each test specimen is also assessed using a non-specific pseudovirus (specificity control) that expresses a non-reactive envelope protein of one or more unrelated viruses (e.g., avian influenza virus).

The testing and validation for the Wuhan-Hu-1 (ancestral) virus and all variant pseudoviruses was performed at Monogram Biosciences in South San Francisco, CA, USA. Validation for the Wuhan-Hu-1 virus included accuracy, repeatability, intermediate precision, linearity, specificity/selectivity, sensitivity, and stability utilizing pooled sera from high-titer, intermediate-titer, and low-titer pooled convalescent SARS-CoV-2 sera, as well as historical negative samples collected in the year 2017 (prior to SARS-CoV-2 circulation). Amino acid changes in variant of concern pseudoviruses are outlined in Supplemental Table 5.

**Analysis populations**

The analyses in this manuscript are restricted to participants in the fully vaccinated analysis set (FVS) with symptomatic reverse transcriptase-polymerase chain reaction (RT-PCR)-confirmed SARS-CoV-2 infection ≥15 days after second dose primary series AZD1222 or placebo who provided samples during the 28-day illness period.

The FVS includes all participants in the full analysis set who were seronegative at baseline, received two doses of study intervention, and who remained on-study 15 days after their second dose without having had a prior SARS-CoV-2 RT-PCR- positive confirmed COVID-19 infection. The safety analysis set consisted of all participants who received at least one dose of study intervention. Erroneously-treated participants who received one dose of active study intervention and one dose of saline placebo were accounted for in this analysis set by assigning them to the study intervention they actually received. A participant who had on one or several occasions received active study intervention was classified as active for all summaries, including summaries by dose. The immunogenicity analysis (IAS) population included all participants in the safety analysis set who had no protocol deviations judged to have the potential to interfere with the generation or interpretation of an immune response.

The participant demography (Supplemental Table 2) comprises all study participants in the IAS who had a positive SARS-CoV-2 RT-PCR result confirmed by the endpoint adjudication committee (AZD122 *n*=177; placebo *n*=203). Illness visit e-Diary summary data (Supplemental Table 3) are from study participants in the FVS only and comprise data from the first set of illness visits associated with a positive SARS-CoV-2 RT-PCR result (AZD1222 *n*=122; placebo *n*=148).

**Censoring implications for non-study COVID-19 vaccines**

For ethical reasons, study participants could be unblinded and receive non-study COVID-19 vaccination once available through emergency-use authorizations. Participants were initially censored for all immunogenicity endpoints at the date of receipt of non-study COVID-19 vaccination, such that data from all subsequent visits was excluded from derivations and all by-visit summaries to provide comprehensive information on durability of immunogenicity post-vaccination (1).

As outlined in Sobieszczyk *et al*. (2), censoring criteria were revised following the observation of increasing levels of anti-SARS-CoV-2 spike-binding and neutralizing antibodies on the placebo arm. Participants in both study arms thus were censored at the earliest date of non-study COVID-19 vaccination, excluding date of unblinding for the AZD1222 arm, and including the date of unblinding for placebo, whichever occurred first, with the aim of excluding effects of unreported non-study COVID-19 vaccinations in the placebo arm. Participants were only unblinded once they became eligible to receive a non-study vaccine and generally participants received their non-study vaccination shortly thereafter.

**SARS-CoV-2 genomic assessments**

SARS-CoV-2 spike protein sequences were assessed by next-generation sequencing (NGS) using the GenoSure SARS-CoV-2 spike NGS assay (Monogram Biosciences, South San Francisco, CA, USA) and assessed at a consensus allele fraction of ≥25%. A spike-only version of Pangolin COVID-19 lineage assigner (https://github.com/aineniamh/hedgehog, version 1.0.2) (3) was used to classify SARS-CoV-2 sequences to current Pango lineages (v.1.2.6) (4) or sets of lineages. Additional supportive SARS-CoV-2 whole genome sequencing analyses were performed on participant saliva samples using the Illumina COVIDSeq Test and software (San Diegeo, CA, USA) (1).

**Serology analyses**

Serum samples allowed to clot during a 30 minute incubation at room temperature. Samples were centrifuged at 1,300xg for 15 minutes at room temperature within 1 hour of collection. Cleared serum stored at –70⁰C prior to analysis.

Serum anti-SARS-CoV-2 spike-binding and nucleocapsid IgG antibody titers were tested at PPD^®^ in a validated multiplex electrochemiluminescence serology assay using the MSD V-PLEX^®^ SARS-CoV-2 Panel 2 IgG as outlined in (5) in its own scale. As outlined in Wilkins *et al*. (5), a bridging experiment was performed to convert spike-binding units in arbitrary units per milliliter (AU)/mL to the WHO international standard (National Institute for Biological Standards and Control [NIBSC 20/136) binding units (BAU/mL). The following formula may be applied to convert spike-binding titers from AU/mL to BAU/mL: (BAU/mL) = AU/mL * 0.00645. Therefore, 1000 BAU/mL = 155,039 AU/mL.

nAbs were assessed in a validated lentivirus-based SARS-CoV-2 pseudovirus assay (Monogram Biosciences, South San Francisco, CA, USA) as described previously (1). nAbs titers are reported as the reciprocal of the serum dilution conferring ID_50_ of pseudovirus infection. Calibration factors to enable conversion from ID50 to the WHO international standard (NIBSC 20/136) in International units (IU/mL) were derived in a calibration study. The following formula may be applied to convert nAb titers from ID50 to IU/mL: (IU/mL) = ID50 * 0.1428. Therefore, 1000 IU/mL = 7001.3 ID50.

**ICS Assay**

Peripheral blood mononuclear cells were thawed and rested overnight before being stimulated with indicated peptide pools (JPT Peptide Technologies) for 6 hours at 37°C with 5% CO_2_. Spike peptides for ancestral and Omicron BA.1 variant were divided equally into two pools (S1 and S2), and reported responses are the combined reactivity to both peptide pools.

Following stimulation, cells were washed and stained with viability dye for 20 minutes at room temperature, followed by surface stain for 20 minutes at room temperature, cell fixation, and permeabilization using the eBioscience FoxP3/Transcription Factor Staining Buffer Set (ThermoFisher catalog # 00-5523-00) for 45 minutes at room temperature, and then intracellular stain for 30 minutes at room temperature. Titration of all antibodies included in the study was performed prior to the analysis of clinical specimens. See Supplemental Table 6 for a complete list of antibodies used in this analysis. Upon completion of staining, cells were collected on a BD FACSymphony A5 Flow Cytometer. Samples were invalidated if <10,000 live CD3+ T cells were collected.

**Flow cytometry gating strategy**

We have previously described our gating strategy (6). Samples were analyzed using FlowJo 10.6.2. Anomalous “bad” events were separated from “good” events using FlowAI (7). “Good events” were used for all downstream gating. Individual cytokines were plotted on the Y-axis versus CD69+ cells on the X-axis, and only CD69+ events were used to determine positive responses. No cytokine-positive responses were detected above background in the CD69-gate. All antigen-specific cytokine frequencies are reported after background subtraction of identical gates from the same sample incubated with negative control stimulation (DMSO). Responder threshold was set on the two-fold absolute median deviation of T cell interferon gamma (IFNγ) signal in all DMSO controls.

**Supplemental figures:**


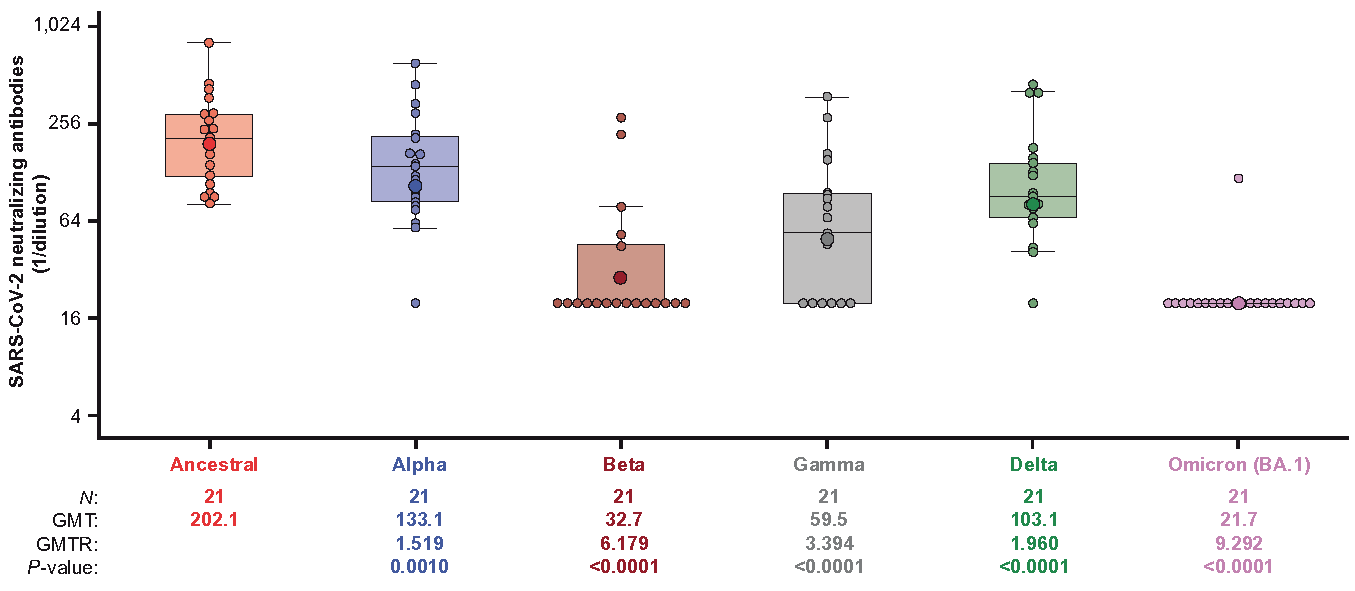


**Supplemental Figure 1.** **nAb responses 28 days post-AZD1222 primary-series vaccination are consistent with expected immune-evasive properties of SARS-CoV-2 Variants of Concern.** Box and whisker plots of the neutralizing activity from pooled AZD1222 vaccinated donor serum (*n*=21 pools collected from 210 donors) collected approximately 28 days post-second dose AZD1222 vaccination against the ancestral SARS-CoV-2 reference pseudovirus and 5 pseudotyped variants of concern. Geometric mean titers of anti-SARS-CoV-2 nAb titers against variants of concern as determined by pseudovirus neutralization assay. The bottom and top edges of the box indicate the first and third quartiles, the difference is the IQR, the line inside the box is the median, and the marker inside the box is the mean. The whiskers that extend from the box indicate the minimum and maximum after removing outliers (i.e., datapoints >1.5 x IQR from the box). Geometric mean titer ratio is calculated as the ratio between the titer value of the ancestral SARS-CoV-2 compared to the titer value of variant. *P*-value is based on a paired t-test comparing the variant to ancestral SARS-CoV-2 on log base 2 antibody levels. Lower limit of quantification (LLoQ) = 40 ID_50_. 50% of LLoQ =20 ID_50_.


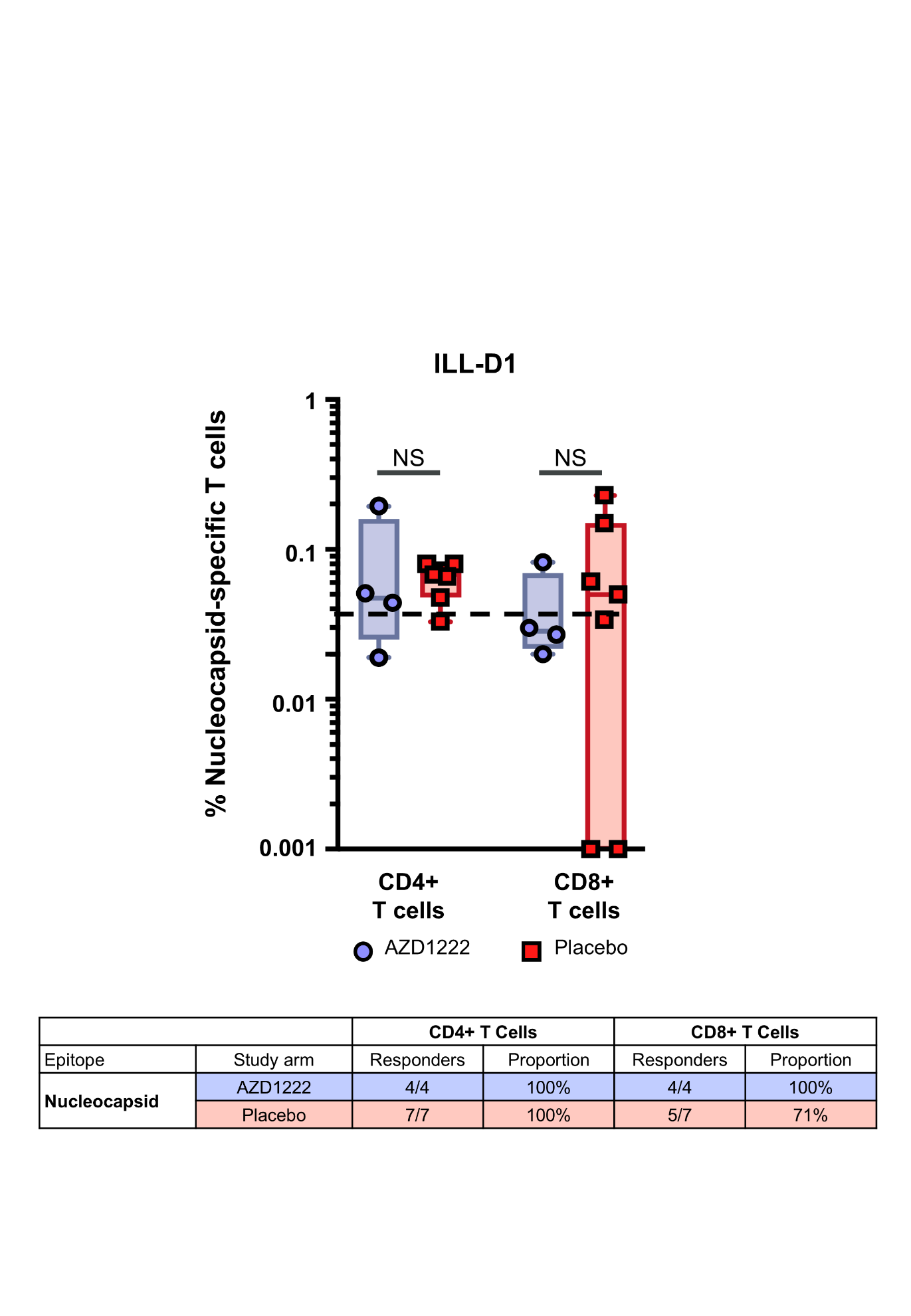


**Supplemental Figure 2. Nucleocapsid-specific CD4+ and CD8+ T cells responses in AZD1222 vaccinees and placebo recipients.** Illness visit day 1 frequencies of nucleocapsid-specific CD4+ and CD8+ T-cell responses in vaccinees and placebo recipients. Boxes represent median values within each group and symbols represent each participant. Statistical evidence between groups were determined by two-tailed Mann-Whitney tests. Not significant (NS), *P*>0.05.


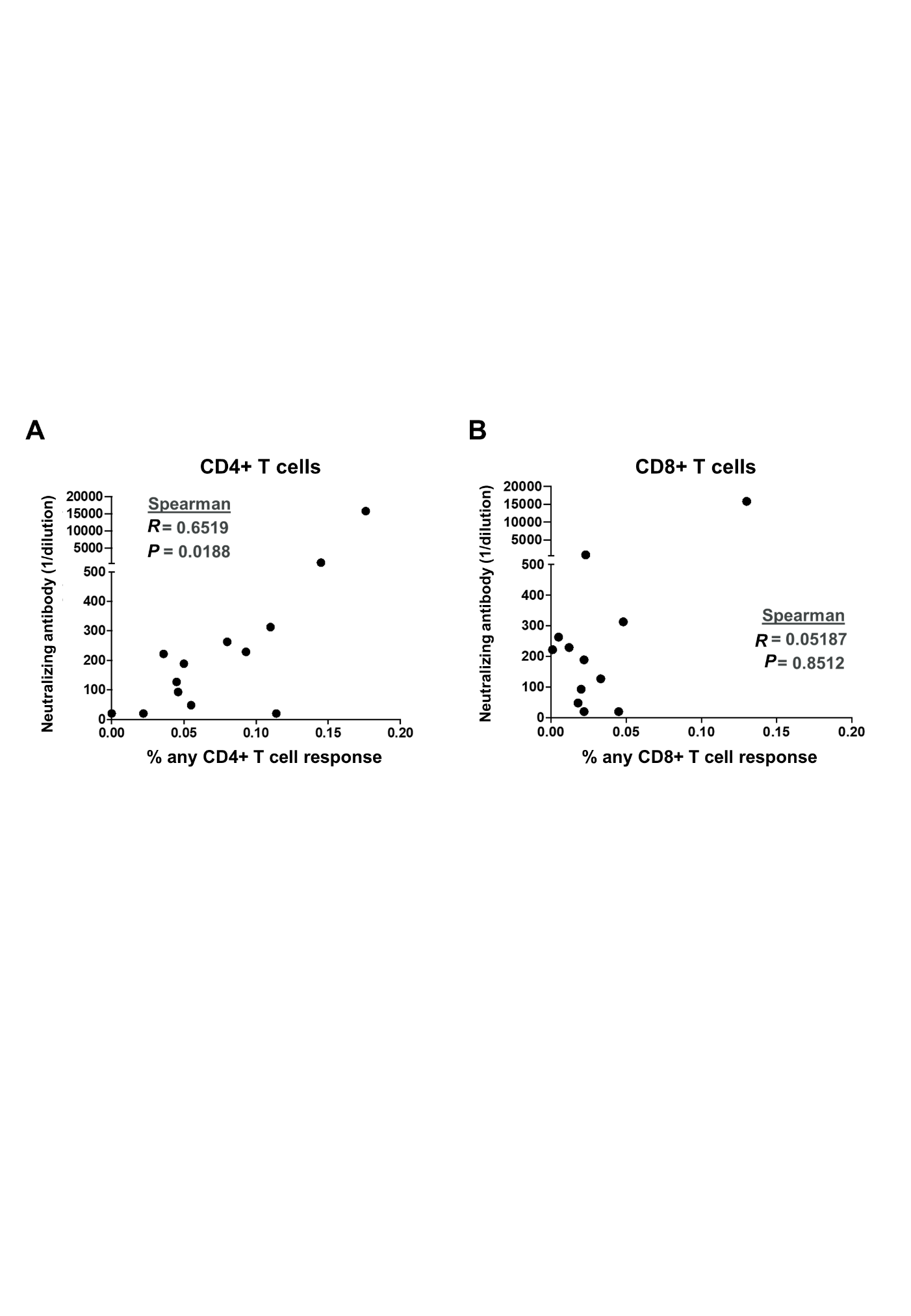


**Supplemental Figure 3. Illness visit day 1 CD4+ T-cell responses correlate with Illness Day 1 nAb titers.** Scatterplot analysis depicting correlations between nAb titers (y-axes) with spike-specific CD4+ **(A)** and CD8+ **(B)** T cells (x-axes) upon breakthrough infection in vaccinees and placebo recipients at the first illness visit.


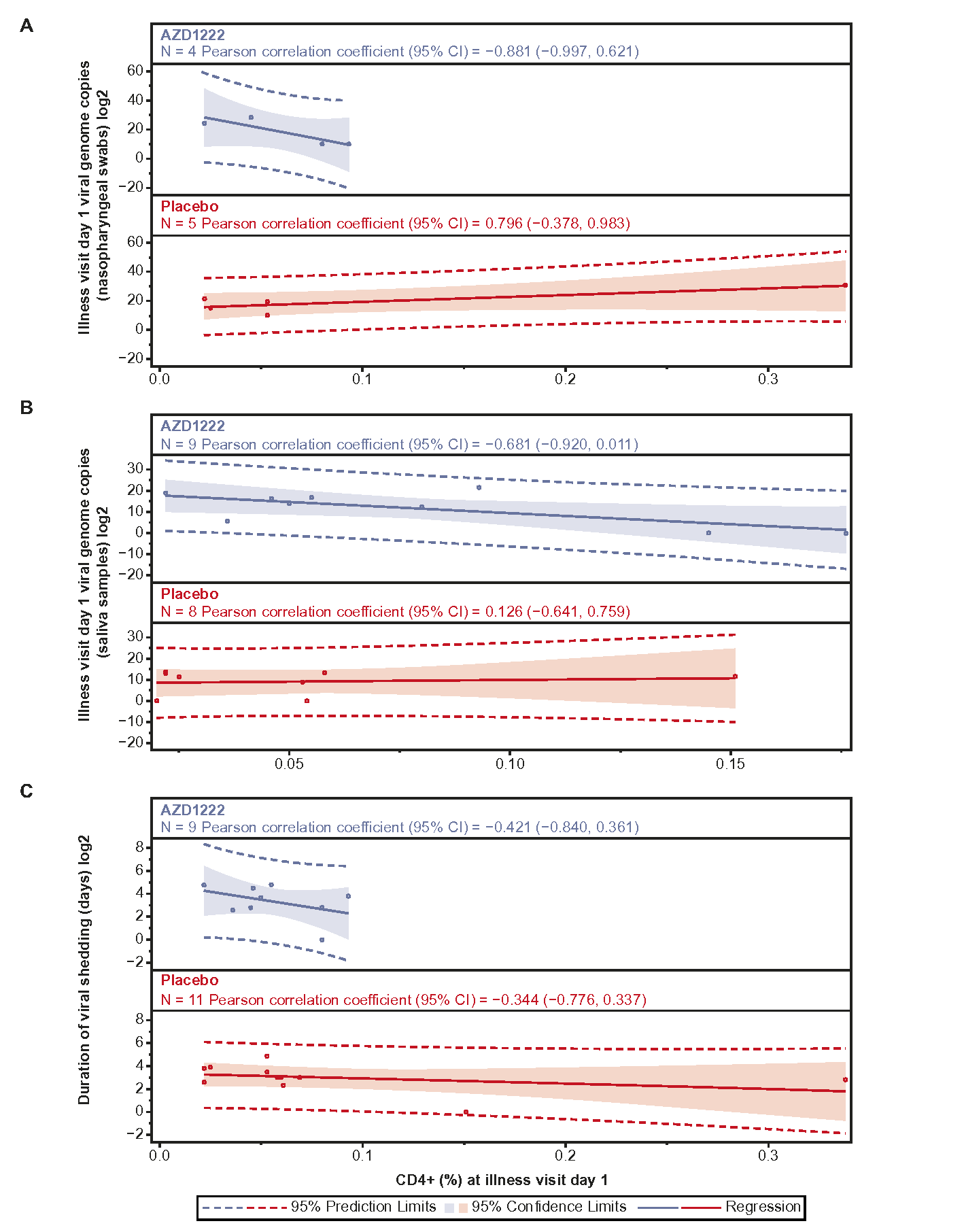


**Supplemental Figure 4.** **Illness visit day 1 CD4+ T cell levels moderately correlate with SARS-CoV-2 virologic outcomes.** Scatterplot analysis depicting the relationship between (**A**) SARS-CoV-2 viral load in nasopharyngeal swabs, (**B**) SARS-CoV-2 viral load in saliva samples, and (**C**) duration of viral shedding (y-axes) and illness visit day 1 CD4+ T cells (x-axes) in vaccinees and placebo recipients. Blue and red shading denotes 95% confidence limits. Dotted line denotes 95% prediction limits.

**Supplemental tables:**

| **Supplemental Table 1. Demographics of fully vaccinated participants who provided serum samples for exploratory immunogenicity analyses.** | | | | | |
| --- | --- | --- | --- | --- | --- |
| **Characteristic** | | **Statistics** | | **AZD1222 (*n* = 210)** | |
| **Age (years)** | | Mean (SD) | | 50.13 (16.376) | |
|  | | Median (Min, Max) | | 52.50 (18.0, 90.0) | |
| **Sex, *n* (%)** | n | | 210 | |  |
| Female | n (%) | | 134 (63.8) | |  |
| Male | n (%) | | 76 (36.2) | |  |
| **Ethnicity, *n* (%)** |  | |  | |  |
| Hispanic or Latino | n (%) | | 17 (8.1) | |  |
| Not Hispanic or Latino | n (%) | | 186 (88.6) | |  |
| Not reported | n (%) | | 6 (2.9) | |  |
| Unknown | n (%) | | 1 (0.5) | |  |
| **Race, *n* (%)** |  | |  | |  |
| Asian | n (%) | | 11 (5.2) | |  |
| American Indian or Alaska Native | n (%) | | 3 (1.4) | |  |
| Black or African American | n (%) | | 13 (6.2) | |  |
| Unknown | n (%) | | 1 (0.5) | |  |
| White | n (%) | | 180 (85.7) | |  |
| Not reported | n (%) | | 2 (1.0) | |  |
| **Serostatus at baseline, *n* (%)** | n | | 210 | |  |
| Negative | n (%) | | 200 (95.2) | |  |
| Positive | n (%) | | 5 (2.4) | |  |
| Missing | n (%) | | 1 (0.5) | |  |
| Not Done | n (%) | | 4 (1.9) | |  |
| **COVID-19 Co-morbidities at baseline, *n* (%)** |  | |  | |  |
| Yes | n (%) | | 111 (52.9) | |  |
| No | n (%) | | 99 (47.1) | |  |

| **Supplemental Table 2. Demographics of participants with illness visits (fully vaccinated analysis set).** | | | |
| --- | --- | --- | --- |
|  | **AZD1222**  **(*n* = 177)** | **Placebo**  **(*n* = 203)** | **Total**  **(*N* = 380)** |
| **Sex and age** |  |  |  |
| Female, *n* (%) | 70 (39.5) | 75 (36.9) | 145 (38.2) |
| Male, *n* (%) | 107 (60.5) | 128 (63.1) | 235 (61.8) |
| Mean age (SD) | 41.61 (13.73) | 46.47 (14.71) | 44.21 (14.45) |
| Median age (range) | 42.0 (18–85) | 46.0 (18–83) | 44.0 (18–85) |
| **Age, *n* (%)** |  |  |  |
| ≥18 to <65 years | 167 (94.4) | 181 (89.2) | 348 (91.6) |
| ≥65 to <75 years | 7 (4.0) | 20 (9.9) | 27 (7.1) |
| ≥75 years | 3 (1.7) | 2 (1.0) | 5 (1.3) |
| **Ethnicity, *n* (%)** |  |  |  |
| Hispanic or Latinx | 71 (40.1) | 65 (32.0) | 136 (35.8) |
| Not Hispanic or Latinx | 104 (58.8) | 136 (67.0) | 240 (63.2) |
| Not reported | 2 (1.1) | 2 (1.0) | 4 (1.1) |
| **Race, *n* (%)** |  |  |  |
| Multiple^a^ | 11 (6.2) | 14 (6.9) | 25 (6.6) |
| Asian | 6 (3.4) | 4 (2.0) | 10 (2.6) |
| Black or African American | 7 (4.0) | 16 (7.9) | 23 (6.1) |
| American Indian or Alaska Native | 30 (16.9) | 21 (10.3) | 51 (13.4) |
| Native Hawaiian or pacific islander | 2 (1.1) | 0 | 2 (0.5) |
| White | 121 (68.4) | 144 (70.9) | 265 (69.7) |
| Not reported | 0 | 4 (2.0) | 4 (1.1) |
| **Country, *n* (%)** |  |  |  |
| USA | 123 (69.5) | 165 (81.3) | 288 (75.8) |
| Chile | 11 (6.2) | 10 (4.9) | 21 (5.5) |
| Peru | 43 (24.3) | 28 (13.8) | 71 (18.7) |
| **COVID-19 comorbidities^b^ *n* (%)** |  |  |  |
| Yes | 93 (52.5) | 126 (62.1) | 219 (57.6) |
| No | 84 (47.5) | 77 (37.9) | 161 (42.4) |
| **Exposure risk to COVID-19 per OSHA Categories *n* (%)** |  |  |  |
| Very high exposure risk | 16 (9.0) | 12 (5.9) | 28 (7.4) |
| High exposure risk | 32 (18.1) | 31 (15.3) | 63 (16.6) |
| Medium exposure risk | 74 (41.8) | 94 (46.3) | 168 (44.2) |
| Lower exposure risk (caution) | 47 (26.6) | 57 (28.1) | 104 (27.4) |
| Missing | 8 (4.5) | 9 (4.4) | 17 (4.5) |
| ^a^Participants who reported more than one race are reported under 'Multiple'. ^b^Conditions which place subject at high risk for acquisition or more severe COVID-19 disease defined as per (1).  OSHA, Occupational Safety and Health Administration. | | | |

| **Supplemental Table 3. Incidence and duration of self-reported COVID-19 symptoms.** Data from illness e-Diaries throughout the illness period by participant age. | | | | | | | |
| --- | --- | --- | --- | --- | --- | --- | --- |
|  | **Statistic** | **AZD1222**  **(*N* = 17,617)** | | | **Placebo**  **(*N* = 8,528)** | | |
| **Participant age** | Years | 18–<65 | ≥65 | Overall | 18–<65 | ≥65 | Overall |
| **Number of participants with illness visits** | *n* | 114 | 8 | 122 | 131 | 17 | 148 |
| **Fever** |  |  |  |  |  |  |  |
| Number and percentage of participants with symptom | *n* (%) | 2 (1.8) | - | 2 (1.6) | 19 (14.5) | 4 (23.5) | 23 (15.5) |
| Duration of symptom (days) | Mean (SD) | 2.5 (2.12) | - | 2.5 (2.12) | 1.9 (1.41) | 1.8 (0.96) | 1.9 (1.32) |
|  | Median (range) | 2.5 (1–4) | - | 2.5 (1–4) | 2.0 (1–7) | 1.5 (1–3) | 2.0 (1–7) |
| **Shortness of breath** |  |  |  |  |  |  |  |
| Number and percentage of participants with symptom | *n* (%) | 18 (15.8) | - | 18 (14.8) | 22 (16.8) | 6 (35.3) | 28 (18.9) |
| Duration of symptom (days) | Mean (SD) | 1.4 (0.92) | - | 1.4 (0.92) | 1.9 (1.63) | 1.0 (0.00) | 1.7 (1.49) |
|  | Median (range) | 1.0 (1–4) | - | 1.0 (1–4) | 1.5 (1–8) | 1 (1–1) | 1.0 (1–8) |
| **Difficulty breathing** |  |  |  |  |  |  |  |
| Number and percentage of participants with symptom | *n* (%) | 46 (40.4) | 1 (12.5) | 47 (38.5) | 63 (48.1) | 8 (47.1) | 71 (48.0) |
| Duration of symptom (days) | Mean (SD) | 3.1 (2.77) | 1.0 (-) | 3.1 (2.76) | 4.0 (4.13) | 3.9 (4.36) | 4.0 (4.12) |
|  | Median (range) | 2.0 (1–12) | 1.0 (1–1) | 2.0 (1–12) | 2.0 (1–20) | 2.5 (1–14) | 2.0 (1–20) |
| **Chills** |  |  |  |  |  |  |  |
| Number and percentage of participants with symptom | *n* (%) | 43 (37.7) | 1 (12.5) | 44 (36.1) | 67 (51.1) | 10 (58.8) | 77 (52.0) |
| Duration of symptom (days) | Mean (SD) | 1.6 (1.05) | 1.0 (-) | 1.6 (1.04) | 2.9 (2.60) | 1.9 (0.88) | 2.8 (2.46) |
|  | Median (range) | 1.0 (1–6) | 1.0 (1–1) | 1.0 (1–6) | 2.0 (1–13) | 2.0 (1–3) | 2.0 (1–13) |
| **Cough** |  |  |  |  |  |  |  |
| Number and percentage of participants with symptom | *n* (%) | 45 (39.5) | 1 (12.5) | 46 (37.7) | 64 (48.9) | 4 (23.5) | 68 (45.9) |
| Duration of symptom (days) | Mean (SD) | 2.2 (2.59) | 1.0 (-) | 2.2 (2.57) | 3.2 (4.03) | 3.5 (2.38) | 3.2 (3.94) |
|  | Median (range) | 1.0 (1–15) | 1.0 (1–1) | 1.0 (1–15) | 2.0 (1–24) | 2.5 (2–7) | 2.0 (1–24) |
| **Fatigue** |  |  |  |  |  |  |  |
| Number and percentage of participants with symptom | *n* (%) | 89 (78.1) | 7 (87.5) | 96 (78.7) | 118 (90.1) | 16 (94.1) | 134 (90.5) |
| Duration of symptom (days) | Mean (SD) | 4.7 (4.18) | 4.3 (3.45) | 4.7 (4.12) | 6.6 (5.85) | 7.6 (6.56) | 6.7 (5.92) |
|  | Median (range) | 3.0 (1–16) | 3.0 (1–10) | 3.0 (1–16) | 5.0 (1–25) | 6.5 (1–25) | 5.0 (1–25) |
| **Muscle aches** |  |  |  |  |  |  |  |
| Number and percentage of participants with symptom | *n* (%) | 62 (54.4) | 5 (62.5) | 67 (54.9) | 97 (74.0) | 13 (76.5) | 110 (74.3) |
| Duration of symptom (days) | Mean (SD) | 3.0 (2.77) | 1.0 (0.00) | 2.9 (2.72) | 3.9 (3.69) | 4.2 (3.56) | 3.9 (3.66) |
|  | Median (range) | 2.0 (1–16) | 1.0 (1–1) | 2.0 (1–16) | 3.0 (1–19) | 3.0 (1–13) | 3.0 (1–19) |
| **Body aches** |  |  |  |  |  |  |  |
| Number and percentage of participants with symptom | *n* (%) | 62 (54.4) | 3 (37.5) | 65 (53.3) | 97 (74.0) | 12 (70.6) | 109 (73.6) |
| Duration of symptom (days) | Mean (SD) | 3.1 (2.84) | 1.3 (0.58) | 3.0 (2.80) | 3.9 (3.81) | 4.8 (3.49) | 4.0 (3.77) |
|  | Median (range) | 2.0 (1–16) | 1.0 (1–2) | 2.0 (1–16) | 3.0 (1–19) | 4.0 (1–13) | 3.0 (1–19) |
| **Headache** |  |  |  |  |  |  |  |
| Number and percentage of participants with symptom | *n* (%) | 70 (61.4) | 6 (75.0) | 76 (62.3) | 108 (82.4) | 15 (88.2) | 123 (83.1) |
| Duration of symptom (days) | Mean (SD) | 3.9 (3.15) | 3.3 (2.50) | 3.9 (3.09) | 4.7 (4.14) | 2.9 (2.63) | 4.5 (4.02) |
|  | Median (range) | 3.0 (1–16) | 2.5 (1–8) | 3.0 (1–16) | 3.5 (1–22) | 2.0 (1–10) | 3.0 (1–22) |
| **New loss of smell** |  |  |  |  |  |  |  |
| Number and percentage of participants with symptom | *n* (%) | 71 (62.3) | 3 (37.5) | 74 (60.7) | 83 (63.4) | 5 (29.4) | 88 (59.5) |
| Duration of symptom (days) | Mean (SD) | 3.9 (4.20) | 4.0 (2.65) | 3.9 (4.14) | 5.5 (4.99) | 1.6 (1.34) | 5.3 (4.94) |
|  | Median (range) | 2.0 (1–22) | 3.0 (2–7) | 2.5 (1–22) | 4.0 (1–21) | 1.0 (1–4) | 4.0 (1–21) |
| **New loss of taste** |  |  |  |  |  |  |  |
| Number and percentage of participants with symptom | *n* (%) | 54 (47.4) | 2 (25.0) | 56 (45.9) | 70 (53.4) | 4 (23.5) | 74 (50.0) |
| Duration of symptom (days) | Mean (SD) | 4.0 (4.18) | 2.5 (0.71) | 3.9 (4.11) | 4.6 (4.53) | 2.8 (2.06) | 4.5 (4.44) |
|  | Median (range) | 3.0 (1–22) | 2.5 (2–3) | 3.0 (1–22) | 3.0 (1–21) | 2.5 (1–5) | 3.0 (1–21) |
| **Sore throat** |  |  |  |  |  |  |  |
| Number and percentage of participants with symptom | *n* (%) | 55 (48.2) | 4 (50.0) | 59 (48.4) | 75 (57.3) | 9 (52.9) | 84 (56.8) |
| Duration of symptom (days) | Mean (SD) | 3.0 (2.74) | 3.8 (3.20) | 3.1 (2.75) | 3.7 (3.55) | 3.6 (2.46) | 3.7 (3.44) |
|  | Median (range) | 2.0 (1–15) | 3.5 (1–7) | 2.0 (1–15) | 2.0 (1–20) | 3.0 (1–8) | 2.0 (1–20) |
| **Congestion** |  |  |  |  |  |  |  |
| Number and percentage of participants with symptom | *n* (%) | 102 (89.5) | 6 (75.0) | 108 (88.5) | 116 (88.5) | 14 (82.4) | 130 (87.8) |
| Duration of symptom (days) | Mean (SD) | 5.3 (4.41) | 5.8 (4.67) | 5.3 (4.40) | 7.0 (5.78) | 8.3 (8.28) | 7.1 (6.07) |
|  | Median (range) | 4.0 (1–22) | 5.5 (1–13) | 4.0 (1–22) | 5.0 (1–24) | 5.5. (1–26) | 5.0 (1–26) |
| **Runny nose** |  |  |  |  |  |  |  |
| Number and percentage of participants with symptom | *n* (%) | 85 (74.6) | 5 (62.5) | 90 (73.8) | 97 (74.0) | 13 (76.5) | 110 (74.3) |
| Duration of symptom (days) | Mean (SD) | 4.0 (3.81) | 3.6 (2.07) | 4.0 (3.73) | 5.4 (5.27) | 6.1 (6.46) | 5.5 (5.39) |
|  | Median (range) | 3.0 (1–22) | 4.0 (1–6) | 3.0 (1–22) | 3.0 (1–24) | 5.0 (1–24) | 3.0 (1–24) |
| = symptom not recorded/no incidence |  |  |  |  |  |  |  |

| **Supplemental Table 4. Frequencies of spike-specific CD4+ and CD8+ T-cell responses at ILL-D1 and ILL-D14.** | | | | | |
| --- | --- | --- | --- | --- | --- |
|  | | **CD4+ T cells** | | **CD8+ T cells** | |
| **Vaccine** | **Timepoint (day)** | **Responders** | **Proportion** | **Responders** | **Proportion** |
| AZD1222 | ILL-D1 | 5/6 | 83% | 5/6 | 83% |
|  | ILL-D14 | 6/6 | 100% | 6/6 | 100% |
| Placebo | ILL-D1 | 11/20 | 55% | 6/20 | 30% |
|  | ILL-D14 | 16/20 | 80% | 11/20 | 55% |

| **Supplemental Table 5. Amino acid changes incorporated into variant of concern pseudoviruses.** | | |
| --- | --- | --- |
| **Variant** | |  |
| **WHO label** | **Lineage** | **Spike mutations** |
| Alpha | B.1.1.7 | ΔH69/V70, ΔY145, N501Y, A570D, D614G, P681H, T716I, S982A, D1118H |
| Beta | B.1.351 | L18F, D80A, D215G, ΔL242/A243/L244, K417N, N501Y, E484K, D614G, A701V |
| Gamma | P.1 | L18F, T20N, P26S, D138Y, R190S, K417T, E484K, N501Y, D614G, H655Y, T1027I, V1176F |
| Delta | B.617.2 | T19R, G142D, Δ156-158, L452R, T478K, D614G, P681R, D950N |
| Omicron | BA.1 | A67V, Δ69-70, T95I, G142D, Δ143-145, Δ211-212, ins214EPE, G339D, S371L, S373P, S375F, K417N, N440K, G446S, S477N, T478K, E484A, Q493K, G496S, Q498R, N501Y, Y505H, T547K, D614G, H655Y, N679K, P681H, N764K, D796Y, N856K, Q954H, N969K, L981F |

| **Supplemental Table 6 List of antibodies used for intracellular cytokine staining.** | | | |
| --- | --- | --- | --- |
| **Fluorophore** | **Specificity** | **Clone** | **Stain** |
| FITC | **CD107a** | H4A3 | Surface |
| BB660 | **IL-13** | JES10-5A2 | Intracellular |
| BB700 | **IFNγ** | B27 | Intracellular |
| BB790 | **CD137** | 4B4-1 | Intracellular |
| PE | **IL-4** | MP4-25D2 | Intracellular |
| PE/Dazzle 594 | **CXCR3** | G025H7 | Surface |
| PE/Cy5.5 | **FoxP3** | PCH101 | Intracellular |
| PE/Cy7 | **PD-1** | EH12.2H7 | Surface |
| AF647 | **IL-21** | 3A3-N2.1 | Intracellular |
| AF700 | **CD25** | M-A251 | Surface |
| APC/C7 | **TCRgd** | REA591 | Surface |
| BUV395 | **CCR7** | 150503 | Surface |
| UV-Blue | **Viability** | - | Surface |
| BUV496 | **CD4** | SK3 | Surface |
| BUV563 | **CXCR5** | RF8B2 | Surface |
| BUV661 | **CD196** | 11A9 | Surface |
| BUV737 | **CD28** | 28.2 | Surface |
| BUV805 | **CD8** | SK1 | Surface |
| BV421 | **IL-2** | MQ1-17H12 | Intracellular |
| BV510 | **IL-17A** | BL168 | Intracellular |
| BV570 | **CD45RA** | HI100 | Surface |
| BV605 | **CD154** | 24-31 | Intracellular |
| BV650 | **Ki-67** | B56 | Intracellular |
| BV711 | **CD69** | FN50 | Intracellular |
| BV750 | **TNF** | MAb11 | Intracellular |
| BV786 | **CD3** | Sk7 | Surface |

# References

1. Falsey AR, Sobieszczyk ME, Hirsch I, Sproule S, Robb ML, Corey L, et al. Phase 3 Safety and Efficacy of AZD1222 (ChAdOx1 nCoV-19) COVID-19 Vaccine. *N Engl J Med* (2021);385(25):2348-60. doi: 10.1056/NEJMoa2105290

2. Sobieszczyk ME, Maaske J, Falsey AR, Sproule S, Robb ML, Frenck RW, Jr., et al. Durability of Protection and Immunogenicity of AZD1222 (ChAdOx1 nCoV-19) COVID-19 Vaccine over 6 Months. *J Clin Invest* (2022);132(18):e160565. doi: 10.1172/jci160565

3. O'Toole A, Scher E, Underwood A, Jackson B, Hill V, McCrone JT, et al. Assignment of Epidemiological Lineages in an Emerging Pandemic Using the Pangolin Tool. *Virus Evol* (2021);7(2):veab064. doi: 10.1093/ve/veab064

4. Rambaut A, Holmes EC, O'Toole A, Hill V, McCrone JT, Ruis C, et al. A Dynamic Nomenclature Proposal for Sars-Cov-2 Lineages to Assist Genomic Epidemiology. *Nat Microbiol* (2020);5(11):1403-7. doi: 10.1038/s41564-020-0770-5

5. Wilkins D, Aksyuk AA, Ruzin A, Tuffy KM, Green T, Greway R, et al. Validation and Performance of a Multiplex Serology Assay to Quantify Antibody Responses Following Sars-Cov-2 Infection or Vaccination. *Clin Transl Immunology* (2022);11(4):e1385. doi: 10.1002/cti2.1385

6. Swanson PA, 2nd, Padilla M, Hoyland W, McGlinchey K, Fields PA, Bibi S, et al. AZD1222/ChAdOx1 nCoV-19 Vaccination Induces a Polyfunctional Spike Protein-Specific Th1 Response with a Diverse Tcr Repertoire. *Sci Transl Med* (2021);13(620):eabj7211. doi: 10.1126/scitranslmed.abj7211

7. Monaco G, Chen H, Poidinger M, Chen J, de Magalhaes JP, Larbi A. Flowai: Automatic and Interactive Anomaly Discerning Tools for Flow Cytometry Data. *Bioinformatics* (2016);32(16):2473-80. doi: 10.1093/bioinformatics/btw191
